# Computational study of the separation of regular sphere clusters in high-Mach-number flow

**DOI:** 10.1017/jfm.2026.11180

**Published:** 2026-02-20

**Authors:** Thomas Whalen, Ralf Deiterding, Stuart Jon Laurence

**Affiliations:** 1 Department of Aerospace Engineering, University of Maryland, College Park, MD 20742, USA; 2 School of Engineering, University of Southampton, Boldrewood Campus, Southampton SO16 7QF, UK

**Keywords:** flow-structure interactions, high-speed flow, hypersonic flow

## Abstract

A coupled computational-fluid-dynamics/finite-element methodology is implemented to investigate the free aerodynamic separation of clusters of equally sized spheres arranged in regular configurations in Mach-20 flow, representing an idealized meteoroid-fragmentation scenario. The regular nature of the initial agglomeration geometries – touching sphere pairs, tetrahedral four-sphere arrangements and face-centred-cubic 13-sphere configurations – allows a systematic exploration of both individual sphere motions and bulk cluster dynamics as the initial orientation is varied. For sphere pairs, a stable lifting configuration arises when the spheres are in contact in a skewed configuration, a phenomenon that can also emerge in the more populous clusters. In the tetrahedral survey, comprising 38 initial orientations, shock surfing of downstream bodies is found to play a significant role in driving the separation dynamics. Despite substantial variations in detailed sphere motions with initial orientation, the trajectory type and final lateral velocity collapse reasonably well with the initial polar angle of the sphere within the cluster. Indices describing the bluntness and asymmetry of the initial configuration are introduced and correlate well with the collective cluster dynamics, though not always in an intuitive way. For the 13-sphere clusters, the dependency of individual sphere lateral velocities follows a similar trend with initial polar angle to the four-sphere case, suggesting that a simplified separation model may be possible for such configurations. The influence of the initial cluster bluntness on the bulk dynamics is somewhat reduced, however, indicating a tendency towards more homogeneous separation as the cluster population is increased.

## Graphical abstract

**Figure:**
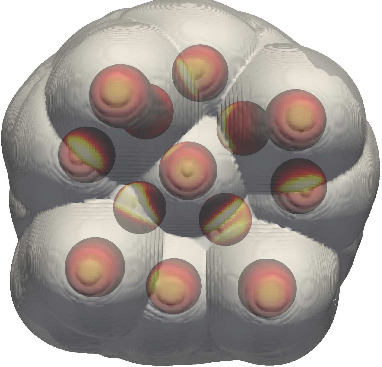

## 1. Introduction

The recent discovery of the potential `city-killer’ asteroid, 2024 YR4, has again highlighted the risks posed to human life and property by the entry of celestial bodies into the Earth’s atmosphere. Such risks are multifaceted, including threats associated with the direct impact of the original object or its fragments at the terrestrial surface, generation of a tsunami for water impacts (Rumpf, Lewis & Atkinson 2017) and energy deposition from the body to the atmosphere, forming a shock wave that can propagate to the surface (Chyba, Thomas & Zahnle 1993). Larger bodies that could result in a mass extinction event (Alvarez *et al.* 1980) typically transit through to the surface unaffected by the atmosphere, but such bodies enter extremely infrequently and can be tracked using available technology. The residual risk to humans from entering meteoroids has thus been shifted to smaller objects (Bland & Artemieva 2003), which cannot be so easily tracked. The extremely high pressures that develop on the front-facing surfaces of these bodies (typically comparable to 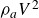 , where 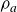 is the atmospheric density and 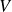 the meteoroid speed relative to the atmosphere) will invariably result in their disruption at some point during atmospheric transit. Aerodynamic interactions between the generated fragments – potentially among other effects (Passey & Melosh 1980) – can greatly increase the ground footprint of those that survive transit, or augment the rate of energy deposition and thus the strength of the shock that propagates to the ground. Therefore, understanding the atmospheric disruption and subsequent separation processes in meteoroid fragmentation is crucial for determining the potential risk posed by such events.

**Figure 1.**
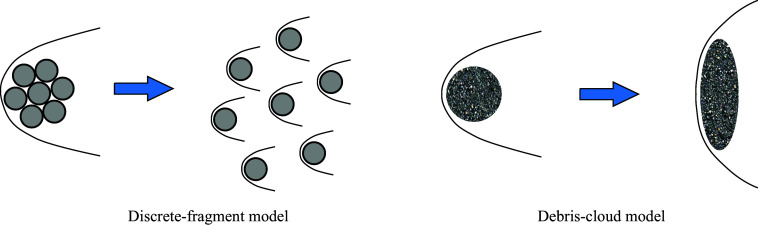
The two approaches typically considered in modelling meteoroid-fragmentation events.

Two approaches have traditionally been taken in investigating the aerodynamic interactions of meteoroid fragments following atmospheric disruption: the `discrete-fragment’ approach, suitable for disruption into a limited number of distinct fragments; and the `debris-cloud’ approach, appropriate for catastrophic fragmentation or the disruption of strengthless, `rubble-pile’ type asteroids (Walsh 2018). These contrasting approaches are summarized in figure 1. The discrete-fragment approach is typified by the binary-fragment model introduced in Passey & Melosh (1980). These authors considered the purely lateral separation of two spherical bodies and derived a final lateral separation velocity of the smaller body, 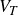 , as
(1.1)

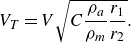

Here, 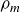 is the density of the meteoroid, 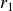 and 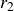 are the two fragment radii and 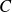 is a ‘constant’: by examining various terrestrial crater fields under the implicit assumption that separation was dominated by such binary interactions, Passey & Melosh (1980) determined 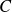 to lie between 0.03 and 2.28. Numerical simulations of the separation of two equal hemicylindrical bodies were subsequently performed by Artem’eva & Shuvalov (1996), and the derived value of 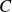 of 0.2 fell within this range. The theoretical and computational modelling of Laurence & Deiterding (2011) and subsequent experiments of Laurence, Parziale & Deiterding (2012), however, showed that the assumption of an exclusively lateral separation, while reasonable for equally sized bodies, is not appropriate for unequal bodies. In particular, the lower ballistic coefficient of the smaller body will typically cause it to be accelerated more quickly downstream, which can result in a phenomenon referred to as ‘shock-surfing’, whereby the smaller body rides the bow shock of the larger body downstream, significantly enhancing its separation velocity.

The `debris-cloud’ approach, in which the fragmented body is treated as a strengthless, liquid-like agglomeration, is exemplified by the semitheoretical `pancake’ models of Chyba *et al.* (1993) and Hills & Goda (1993). Essentially, the aerodynamic forces are assumed to compress the strengthless mass in the streamwise direction and expand it in the lateral direction, increasing the exposed area and thus the rate of energy deposited to the atmosphere, until a terminal radius (some multiple of the initial radius) is reached. Such pancake models have been employed, for example, by Register, Mathias & Wheeler (2017) and McMullan & Collins (2019) in attempts to match light curves of recorded entry events, but in some cases unrealistic expansion ratios (up to seven times the initial radius) have been found to be necessary, whereas Artemieva & Pierazzo (2009) caution against the use of a terminal radius above approximately twice the initial one.

The binary-fragment model just discussed represents the low-population limit of the discrete-fragment approach, while the debris-cloud approach can be considered the other extreme of an effectively infinite fragment-cloud population. The intermediate population regime, however, has received comparatively limited attention. Artemieva & Shuvalov (2001) conducted limited numerical simulations of the separation of 13- and 27-cube clusters using a hydrocode and derived a separation constant of 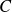 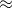 1, though the fidelity and accuracy of these simulations is somewhat unclear. Another example is the study of Park & Park (2020), who performed experiments using a ring-like configuration of up to six spheres and proposed a basic model that would indicate a 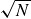 dependence of the mean separation velocity on cluster population, 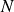 . The somewhat unphysical nature of the parent configuration employed in these experiments, however, does lead to questions regarding its applicability to actual fragmentation events. The need for more accurate models of discrete fragmentation has been highlighted by the observations of Borovicka & Kalenda (2003), who analysed video records of the Morávka fall and derived separation velocities up to an order of magnitude higher than those predicted by existing models.

To help address the deficiencies in our current understanding of high-speed multibody separation, two of the present authors introduced a systematic experimental methodology for studying the aerodynamic separation of populous clusters (Whalen & Laurence 2021), whereby sphere agglomerations were released impulsively into a hypersonic wind-tunnel flow. Preliminary experiments revealed some of the key physics associated with small- to medium-population (up to 36-sphere) clusters. It was found, for example, that the separation process could be divided into two phases: a `primary’ phase, during which the individual separation velocities increased rapidly and almost linearly, and a secondary phase, which was typically characterized by strong subcluster interactions. The ultimate goal of such experimental investigations is to provide a validated statistical description of multibody separation, using random realizations over a specified parameter space. Due to the difficulties in precisely resolving sphere motions early in the separation process, however, these experiments are less well suited to provide a detailed understanding of the governing physics as specific parameters governing the cluster arrangement are varied. Therefore, in parallel with these experimental efforts, we have developed a numerical methodology, combining computational fluid dynamics (CFD) and finite element analysis (FEA), for studying multibody separation. In the present work, we employ this methodology to study the separation of spheres from regular arrangements of two, four and 13 spheres in high-Mach-number flow. The regular nature of the configurations employed allows systematic variation of the relevant geometric parameters and an exploration of their effects on the separation dynamics.

This article is structured as follows. In § 2, the simulation methodology is outlined, including the definition of important quantities, descriptions of the combined CFD–FEA framework employed here and the various configurations simulated, and verification and validation studies. Discussions of the important results for two-sphere, four-sphere and 13-sphere clusters are then provided in §§ 3, 4 and 5, respectively. In the latter two sections, we first discuss general separation characteristics and individual sphere motions, and then proceed to bulk cluster dynamics. Finally, conclusions are drawn in § 6.

## 2. Simulation methodology

### 2.1. Model problem and definitions

The model problem of interest here, intended to represent a meteoroid-fragmentation event, is the separation of a regular cluster of equally sized spheres, released impulsively into a hypersonic flow, through mutual aerodynamic repulsion. Compared with a realistic fragmentation event, the configuration studied here is clearly highly idealized, in the specification both of the parent cluster (regular geometry) and the fragments (spherical and equal sized). This simplification is intentional, however, as it allows a systematic investigation of the problem, and the understanding gained is expected to form a foundation for future studies in which these assumptions can be relaxed. Although ablation effects will be present in a realistic entry situation, the simulations of Artemieva & Shuvalov (2001) have shown these to have a negligible influence on the separation dynamics, and they are thus ignored here. We further restrict our investigation to perfect-gas flows. Although high-temperature effects will be present at the hypervelocity conditions encountered during meteoroid entry (e.g. vibrational excitation, dissociation, ionization) and lead to deviations from perfect-gas behaviour, such effects generally have only a modest influence on pressures in shock-dominated flows (Vincenti & Kruger 1965), and the intersphere pressure will be the dominant effect in driving the cluster dynamics (Laurence & Deiterding 2011).

Before proceeding further, it is useful to define several quantities that will be employed to characterize the separation behaviour of the cluster. The characteristic time scale and velocity are defined as
(2.1)

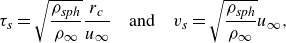

where 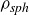 is the sphere density, 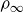 and 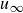 are the free stream density and velocity and 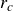 is the initial circumscribed radius of the cluster. The lateral separation velocity of the 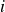 th sphere in the cluster, 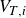 , is referenced to the non-stationary cluster centre of mass (subscript 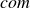 ), and is defined as the magnitude of the component in the 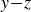 plane according to 
(2.2)

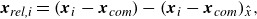





(2.3)

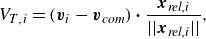

where the 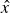 subscript indicates the component in the streamwise direction. The collective lateral separation velocity of the cluster is then defined as
(2.4)

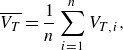

and the relevant non-dimensional quantities characterizing the cluster separation are
(2.5)

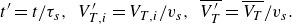




### 2.2. Numerical methods

To determine the dynamics of the spheres within the cluster, a foremost task is computing the flow field generated by the presence of the obstructing bodies, and in particular the pressure distribution over each sphere. The fluid pressure distributions over the spheres, in turn, dictate the aerodynamic forces experienced by each. We note, however, that the complete dynamical behaviour of the bodies will also be influenced by the surface contact that they will inevitably experience while in close proximity at early times, as well as the potential later periods of collision and sustained contact. The situation under investigation thus forms a multiphysics problem, which we approach by coupling a compressible CFD solver to a finite element analysis software for explicit solid mechanics.

A primary challenge of simulating the flow around a group of separating spheres is appropriately modifying the topology of the fluid mesh while maintaining high accuracy in the flow field solution. Previous studies of computational free-flight sphere separation (Laurence *et al.* 2012; Butler *et al.* 2021) have implemented codes specifically designed to handle complex embedded boundaries robustly and adaptively refine the simulation mesh in regions of the flow field identified to contain features such as shock waves. Because many of the dynamically relevant features in the flow over a sphere at hypersonic conditions tend not to be viscous in nature (Laurence, Deiterding & Hornung 2007), we can model the physics approximately with the equations of inviscid flow to reduce computational cost. Although this choice comes at the expense of accuracy in the wake region of a sphere, as will be seen throughout this work (and as can be inferred from earlier work such as Laurence *et al.* 2012), the contribution of wake dynamics to the overall separation behaviour of equal-sphere clusters is minimal.

The fluid solver chosen to compute the unsteady flow fields in this work is Adaptive Mesh Refinement in Object-Oriented C++ (AMROC) (Deiterding 2011), which solves the Euler equations for inviscid flow:
(2.6)

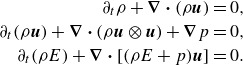

Here 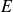 represents the specific total energy, with the pressure determined from the polytropic equation of state, 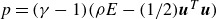 . All solid features are treated as embedded boundaries within a Cartesian mesh (Deiterding 2009), while spatial discretization is formulated in a finite-volume flux-splitting scheme. The MUSCL-Hancock (monotonic upstream-centred scheme for conservation laws-Hancock) reconstruction method with a Min-Mod limiter is implemented via Van Leer flux vector splitting for estimation of numerical flux at cell interfaces. Away from shocks and discontinuities, this semidiscrete formulation provides second-order accuracy, reverting to first order near embedded boundaries across which a ghost-fluid-based interpolation scheme mirrors primitive variables. An explicit Euler time-marching scheme is used throughout. The embedded boundary method in AMROC is a variant of the first-order-accurate ghost-fluid method proposed by Fedkiw *et al.* (1999). Further details of the Cartesian fluid schemes and also verification of the embedded boundary method can be found in Deiterding (2011); for validation comparisons for dynamically moving bodies we refer in particular to Laurence *et al.* (2012).

**Figure 2.**
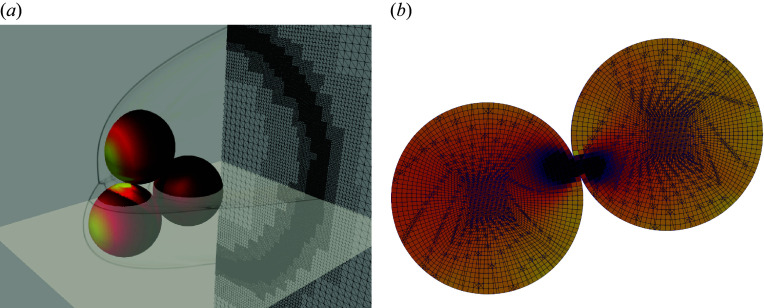
(*a*) Representative computational mesh with three fluid refinement levels showing surface pressure, numerical schlieren and automatically refined mesh capturing the shock. (*b*) Collision of two spheres demonstrating the mesh structure employed and multibody contact capabilities of DYNA3D with cells coloured by principal stress in horizontal direction.

The central advantage of AMROC is its use of a fully parallelized adaptive mesh refinement (AMR) scheme that permits the effective capture of transient flow features associated with moving boundaries. The patch-based approach divides the underlying Cartesian mesh into refinements subsets which are evaluated recursively, with the relative iteration count imposed by a prescribed target Courant–Friedrichs–Lewy (CFL) number. The refinement process is controlled by user-defined gradient thresholds of selected state variables (typically density) and by wall proximity as determined by the level-set function. The parallelized AMR method is equipped with both load-balancing and repartitioning to account for evolving mesh topologies. Figure 2(*a*) provides a demonstration of AMROC’s mesh-refinement capabilities; further details may be found in Deiterding (2011).

A crucially important aspect of AMROC is its verified interface to Lagrangian finite element solvers in a software system called the Virtual Test Facility (VTF). While Deiterding *et al.* (2006) implemented and demonstrated fluid–structure coupling to a volumetric finite element solver, Cirak, Deiterding & Mauch (2007) provide rigorous validation for the fluid–structure coupling of a thin-shell finite element solver, including problems with fracture and fragmentation.

The structural modelling component of the numerical framework here is accomplished using the DYNA3D solver. The DYNA3D solver is an explicit, nonlinear finite-element code commonly used to capture high-speed structural phenomena and allows for a range of material models and contact physics (Hallquist & Jin 2005). A structured grid of regular hexahedral elements is required for the current simulations, for which we employ a three-dimensional butterfly mesh with relaxation of the external cell blocks; this ensures reliable execution for a spherical structural domain, as illustrated by the touching spheres of figure 2(*b*). A critical strength of DYNA3D is its robust and seminal contact detection algorithm (Hallquist, Goudreau & Benson 1985); a global search for proximity between principal surfaces and subsidiary nodes, supported by a detailed contact-checking routine, provides proper treatment of nodal penetration and directional pushback for multibody impacts in dynamic simulation settings. Sliding surface dynamics are computed using standard friction laws, with kinematic and static friction coefficients held constant at 0.5 and 0.7, respectively, in the present implementation. To examine the influence of the choice of friction coefficients, additional simulations were performed with values of 0.1/0.02 (i.e. a very smooth surface) and 1.5/0.9 (a highly roughened surface). For two-sphere separation, the influence on the sphere dynamics was entirely negligible; for four-sphere separation, modest changes in the sphere trajectories were observed, but these were substantially smaller than changes that resulted from variations in the initial cluster orientation. We further assume an elastic material model, although material failure along predefined fault lines would represent a natural extension of our methodology.

Coupling the fluid and structural solvers is accomplished here similarly with VTF routines (cf. Deiterding *et al.* 2006; Cirak *et al.* 2007) and by transmitting to DYNA3D the pressure boundary conditions, to which the principal stresses of boundary cells are equated, and to AMROC the updated geometric boundaries, which determine the kinematics of embedded fluid ghost cells (Deiterding & Wood 2013). Boundary-condition transfer between solvers is updated serially, which increases computational cost in comparison with parallel execution but ensures a higher degree of numerical stability. Computation of the level-set function for imposing embedded wall boundary conditions in the Cartesian AMROC solver is performed by applying the ‘closest point transform’ of Mauch (2003) to the triangulated surface mesh of the solid. Solid and fluid meshes are constructed such that nodal spacing is roughly equivalent on the finest grid level, while the global time-stepping parameter is determined by selecting the minimum of the stress-wave transmission in the solid and target CFL time scales from the fluid. In this study, DYNA3D is run on a single core, whereas AMROC is parallelized across a larger number of processors.

We note that the situation under investigation here, i.e. a fully elastic separation problem lacking significant feedback from structural deformations into the flow field, does not ultimately require treatment of the material response with a finite element analysis, as less complex models (such as the discrete element method of Mishra & Rajamani (1992)) may produce similar results. The FEA-based approach was primarily chosen because AMROC had been earlier coupled to DYNA3D and successful verification and validation for various shock-driven fluid–structure interaction scenarios had already been achieved (Deiterding & Wood 2013), thereby circumventing the need for the development of new coupled computational tools.

### 2.3. Simulation parameters

In all simulations, the fluid is a perfect gas ( 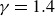 ) with an inflow Mach number of 20. This latter value is somewhat below that typical of meteoroid entry ( 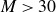 ) but is sufficiently high that the Mach-number independence principle (Anderson 2006) can be expected to hold over the extent of the simulated domain. The ratio of sphere-to-fluid density was set to 1 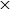 10 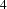 (in dimensional terms, 8000 versus 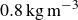 ); structurally, we employ a fully elastic material model with a Young’s modulus of 200 GPa and a Poisson ratio of 0.28 (i.e. values appropriate for iron). In each simulation, the cluster was initially held in place while the inflow velocity was ramped up; once steady state was achieved, the spheres were impulsively released and subsequently allowed to fly freely in response to their experienced forces, consistent with the model problem described above.

**Figure 3.**
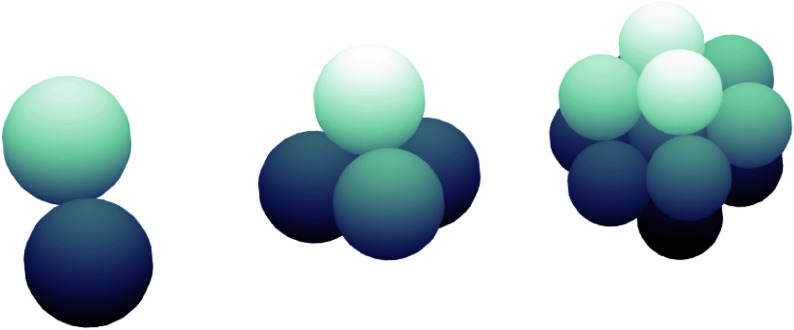
General geometric appearance of two-, four- and 13-sphere close-packed clusters.

**Figure 4.**
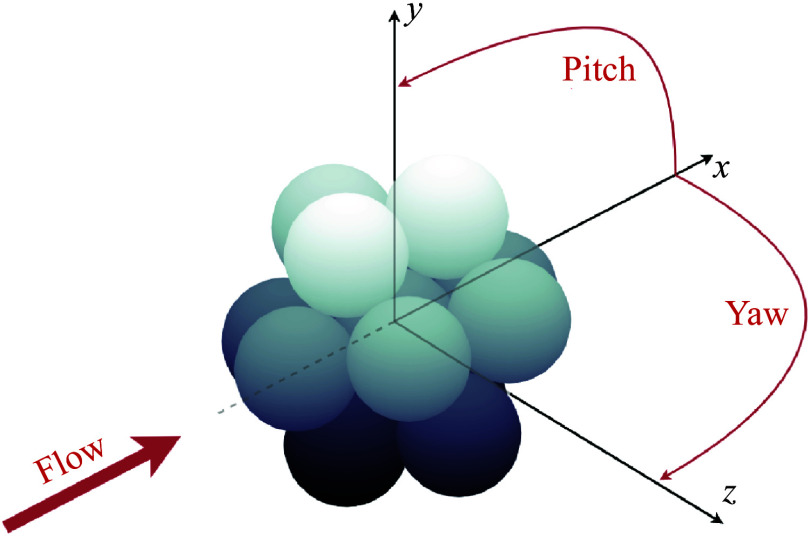
Rendering of the 13-sphere cluster geometry with principal attitude and pitch/yaw angles shown.

Regular clusters of equal-radius spheres with three different populations – two, four and 13 bodies – are examined for various initial orientations in this work, and examples are shown in figure 3. For reference, the sphere radius, 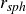 , is set as 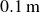 in all cases. For the sphere-pair simulations, the bodies are initially in contact and rotated about their common centre-of-mass by a variety of pitch values. For more populous clusters, we maintain close-packed sphere configurations and vary both the pitch and yaw angles of the cluster from its principal attitude, as shown in figure 4 for a 13-sphere cluster (the exact details of the selected parameter values in each survey will be given in the appropriate subsections of later chapters). Four-sphere clusters are thus formed by positioning the sphere centres at the vertices of a regular tetrahedron, and 13 spheres by constructing two layers of a face-centred cubic lattice. Each structural mesh for the two- and four-sphere clusters contains 2400 surface elements, and the refinement levels of the solid and fluid domains are matched such that the maximum stable time-step size in DYNA3D roughly equates to a target CFL number of 0.8 for time-stepping control in AMROC. Due to the drastically increased computational demands of the 13-sphere cluster simulations, in this case we employ a coarsened mesh and a reduced CFL target of 0.7 to promote the stability of the computations. The exact spatial scales of the fluid domain vary between cluster populations, as does the simulation duration, and this information can be found in table 1 along with the utilized refinement factors. Typical computational times increased from 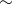 700 CPU hr for two bodies to 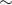 1300 and 4000 CPU hr for the four- and 13-sphere agglomerations, respectively, on a 56-core Dell Precision T7820 workstation. In total, 13 runs were conducted for two spheres, 36 for four spheres and 34 for 13 spheres.

**Table 1.** Simulation parameters.

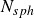	Grid	Base grid	Domain size	Refinement	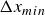	Duration
	(Structure)	(Fluid)	( 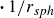 )	Refinement factors	( 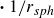 )	( 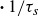 )
2	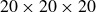	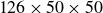	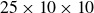	[2, 2, 0]	0.050	9.0
4	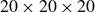	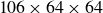	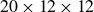	[2, 2, 0]	0.047	5.4
13	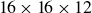	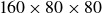	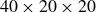	[2, 2, 0]	0.063	6.0

### 2.4. Model verification

To assess the computational reliability of the present numerical model, we performed a grid-refinement study of a specific four-sphere configuration. The initial orientation for this configuration was 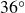 -pitch/ 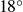 -yaw (the convention for these angles is described at the beginning of § 4); this case was chosen as it exhibited many of the relevant interaction phenomena seen throughout the survey. The verification study consists of four simulations of successively refined fluid and structural domains; in our nomenclature, the numeric simulation labels (Cases 1–4) refer to the increasing degree of overall refinement in each case. Two base-grid resolutions, of dimensions 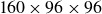 and 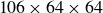 , were used: in the coarsest simulation (i.e. Case 1), the finer base mesh was refined just once by a factor of two, yielding a minimum edge distance, 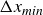 , of 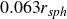 ; while Case 2 achieved a 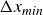 value of 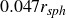 with two refinement passes of the coarse base mesh. Cases 3 and 4 added an extra level of refinement (of factor two) to each of these. With Case 2 representing the baseline resolution, the overall relative refinement levels of the other cases were 75 %, 150 % and 200 %. The solid meshes employed in each case were constructed to match the size of the finest fluid cells, and time-step sizes were automatically adjusted to satisfy a CFL condition of 0.8. All computations were run on a 56-core Dell Precision T7820 workstation: the coarsest simulation required 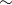 1000 CPU hr to complete and the finest simulation 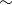 18 000 CPU hr. Details of the grid dimensions, refinement strategies, and execution statistics can be found in table 2.

**Table 2.** Grid refinement study parameters.

Case	Grid	Base grid	Refinement	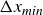	Steps	Cores	CPU
no.	(Structure)	(Fluid)	factors	( 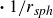 )			[hr]
1	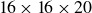	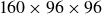	[2, 0, 0]	0.063	44 314	56	1 064
2	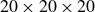	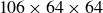	[2, 2, 0]	0.047	55 620	28	1 307
3	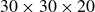	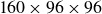	[2, 2, 0]	0.031	83 932	56	7 300
4	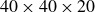	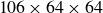	[2, 2, 2]	0.023	111 984	56	18 200

**Figure 5.**
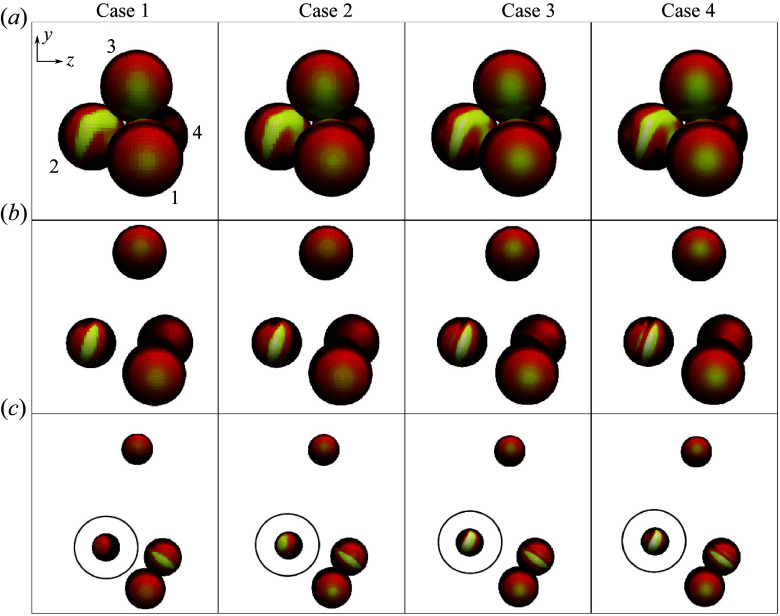
Visualization sequences (looking from directly upstream) of all grid refinement cases in increments of 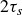 with sphere surfaces coloured by pressure and the trajectory discrepancy of sphere 2 highlighted in (*c*).

Before quantifying the effects of varying resolution, we briefly describe the general behaviour exhibited by the spheres in the chosen configuration. For this arrangement, presented before release in figure 5(*a*), one body lies furthest upstream (sphere 1) and generates a bow shock that impinges on two bodies farther downstream (spheres 2 and 3), while a fourth sphere (sphere 4) is nearly aligned with sphere 1 in the streamwise direction and is therefore mostly shielded from the free stream flow. Following the visualizations of Case 4 (the right-hand column of figure 5), we see that the region of elevated surface pressure inboard on sphere 3 fosters immediate lateral repulsion (vertical, in this case). Sphere 2 experiences a broad band of high pressure near its centre and extending inboard; as it is repelled from the cluster, this irregular impingement pattern develops into the more standard Edney Type-IV shock–shock interaction footprint (Edney 1968), which persists and broadens as the sphere moves downstream along a shock-surfing trajectory. Finally, spheres 1 and 4 engage in paired travel (to be described shortly): sphere 4 remains in contact with and in the aerodynamic shadow of sphere 1 for 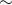 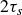 , before being pushed laterally outward from their mutual contact point.

Now qualitatively comparing the highest-resolution Case 4 with the other cases visualized in figure 5, we first note that, while the motions of spheres 1 and 3 remain nearly identical across the grid survey, small discrepancies arise for sphere 4 and larger ones for sphere 2. In particular, although the trajectory of sphere 2 appears to be only weakly affected by reducing the resolution to Case 3, reducing it further leads to a decrease in the duration of shock surfing for this body, with an increased tendency towards entrainment within the shock layer of sphere 1. This is most clearly seen from the band of high pressure from shock impingement on this body at the last time step, which moves from the near the centre of the sphere for Case 4 to the outermost part of the sphere for Case 1. Indeed, the sensitivity of shock-surfing configurations to grid resolution has been already observed in Laurence *et al.* (2012), where it was attributed to a decreased effective bow shock radius generated by the upstream body at higher refinement levels.

**Figure 6.**
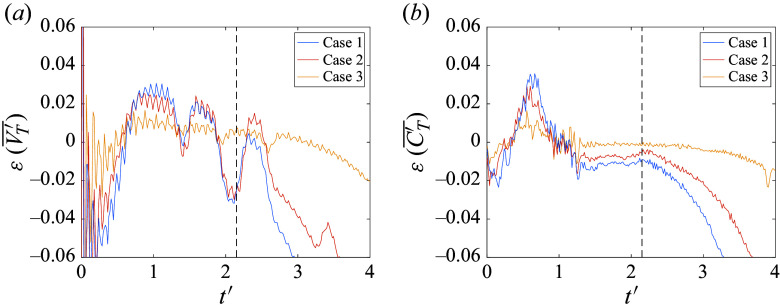
Error in (*a*) mean lateral velocity and (*b*) mean lateral force coefficient relative to finest simulation (Case 4) for Cases 1–3; the end of primary separation phase is indicated with a dashed black line.

In figure 6, we quantify the errors accrued in the mean lateral velocities and force coefficients of the cluster. The lateral force coefficient, 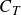 , is defined as 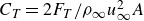 , where 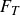 is the lateral force and 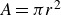 is the projected sphere area. Examining first the mean lateral velocity (figure 6 *a*), the spheres of Case 3 appear to follow trajectories almost identical to those in the finest simulation, with the error remaining within 1 % for the majority of the flight. Cases 1 and 2, on the other hand, show elevated errors that are somewhat obfuscated by the residuals resulting from the series of collisions between spheres 1 and 4. Prior to a substantial divergence of the trajectories at 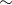 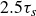 (primarily from sphere 2, as discussed above), errors are limited to 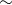 3 %, with no obvious differentiating trends between Cases 1 and 2 (though the lateral velocity errors for Case 1 deviate more sharply thereafter). The errors in the mean lateral force coefficient in figure 6(*b*) give a clearer picture of the purely aerodynamic contribution to these discrepancies. Here, the overall error experienced appears well-correlated to the refinement level of each simulation. Following the positive peak in force error (which is induced by a delayed abatement of repulsive forces on sphere 3), we observe approximately constant errors of 1.0 % for Case 1, 0.6 % for Case 2 and 0.1 % for Case 3 over a duration of approximately 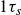 . As with the lateral velocity curves, however, diverging trajectories for the coarser simulations give rise to augmented errors thereafter. Nevertheless, despite the observed inconsistency of the various refinement cases after 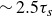 , the dynamical regime of most interest for these simulations is that during which the bodies are in close proximity; this so-called primary separation stage ends at 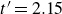 for the present configuration (the criterion for determining this will be described later). Clearly, the sphere trajectories are very nearly converged over this phase in Case 3, but the six-fold increase in computation time over Case 2 renders this choice (and even more so for Case 4) infeasible for parametric survey purposes. Instead, the modest force errors offered by refinement Case 2 ( 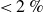 over the primary separation), coupled with its significantly reduced computational expense, make it a suitable choice for the bulk of our numerical investigation. For the largest cluster population ( 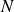 = 13), however, even this level of refinement resulted in a prohibitive computational cost, and the effective resolution was reduced to a level consistent with Case 1. While this may lead to limited accuracy for certain trajectories (particularly those involving shock surfing), we still expect the primary phase to be reasonably well captured at this refinement level.

### 2.5. Experimental validation

**Figure 7.**
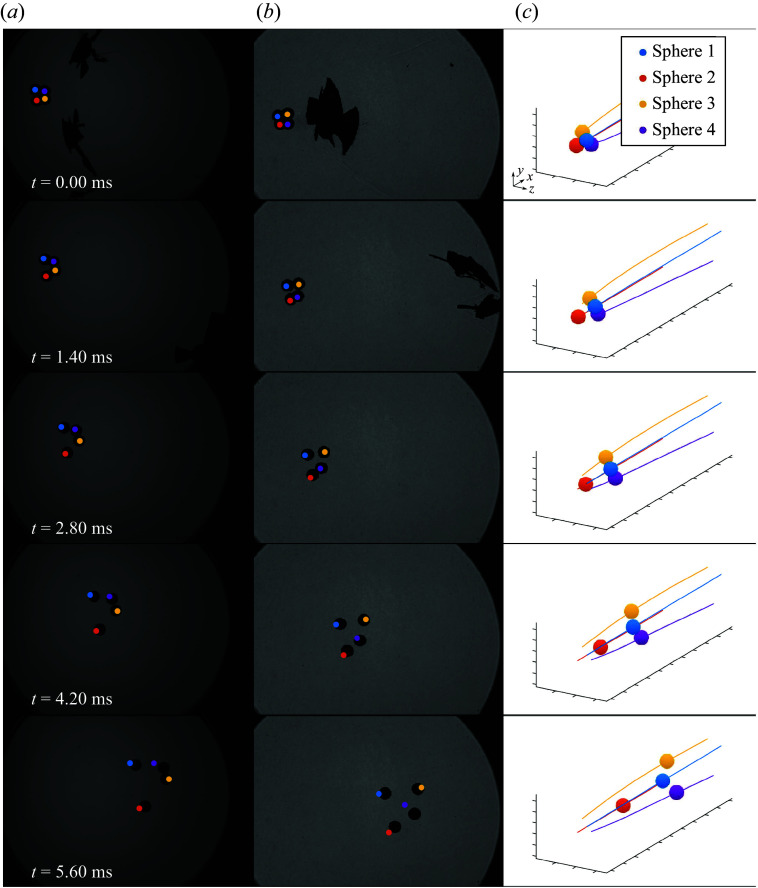
Separation sequence of the four-sphere validation experiment with (*a*) vertical standard camera, (*b*) horizontal shadowgraph camera and (*c*) positional reconstruction. Markers in (*a*) and (*b*) indicate numerical sphere positions.

To provide a further validation of the coupled numerical methodology, we compare results from an experiment involving separating free-flight spheres at Mach 6 with those from a simulation with fully matched conditions. The experiment (Shot 4A from Whalen & Laurence (2021)) features four spheres of diameter 6.35 mm arranged in a tetrahedron (as in the grid-refinement study of the previous subsection), with two leading spheres lying at similar streamwise locations and a pair of roughly aligned trailing spheres. Simulations of clusters with similar orientations produced extended shock surfing, a scenario highly sensitive to relative sphere positions and thus a challenging benchmark test. In figure 7, a sequence of images taken from both below and the side of the wind-tunnel test section (with backlit and shadowgraph set-ups, respectively) depict the sphere motions, along with a three-dimensional reconstruction of the extracted position in figure 7(*c*). Here, 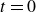 represents the point from which we draw the initial kinematics for the counterpart numerical simulation, chosen such that the shells in which the spheres were initially suspended had separated sufficiently so as not to influence the sphere motions further. As shown in the 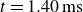 frames, the two leading spheres experience strong repulsive forces and separate laterally almost immediately, which exposes the trailing spheres to the free stream flow. Indeed, in the shadowgraph visualization of 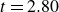 ms, impingement of the shock from sphere 1 on sphere 4 is apparent and the resulting drag augmentation is reflected in the subsequent increased positional separation between the two. It appears that spheres 1 and 2 have ceased aerodynamic interaction by frame 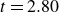 ms, while spheres 3 and 4 remain under the influence of sphere 1 for an extended period of time. From the three-dimensional reconstruction, sphere 3 has begun to fall into the wake of sphere 1, while the shock–shock interaction on sphere 4 moves farther towards its inboard side (see figure 7 *b* for 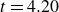 and 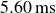 ), indicating an eventual expulsion trajectory.

The complementary simulation (performed with a resolution consistent with verification Case 2 above) exhibits qualitatively similar sphere-separation behaviour, as evidenced in the projected numerical sphere positions in figure 7(*a,b*). Note that the spheres are assigned non-zero initial velocities at 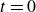 (in each case limited to less than 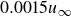 ) to match the experimental conditions at the same time; this was achieved by applying brief impulses to the spheres synchronous with the commencement of the coupled portion of the computation.

**Figure 8.**
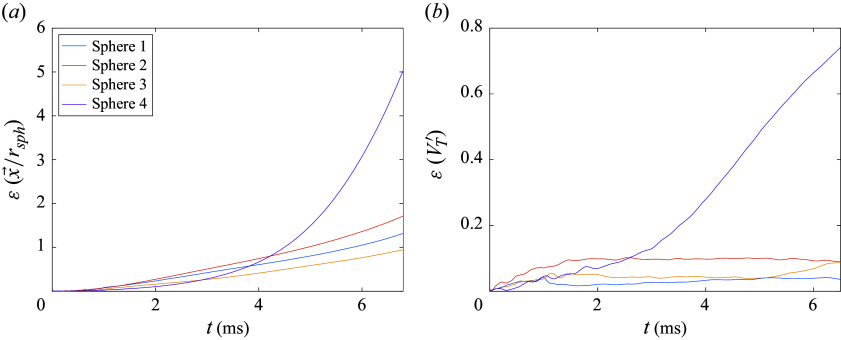
(*a*) Positional error between computation and experiment normalized by the sphere radius and (*b*) error in non-dimensional lateral velocity.

As a quantitative measure of the agreement between the experiment and simulation, we present a comparison of total positional errors (normalized by the sphere radius) in figure 8(*a*) and non-dimensional lateral velocity errors in figure 8(*b*). Most simulated spheres follow the same general paths as in the experiment, remaining within one diameter of the corresponding experimental positions, but sphere 4 shows significant deviation beginning at roughly 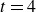 ms. Relative to the experiment, this sphere exhibits reduced displacement in the streamwise direction, while its lateral motion more closely follows that of sphere 1; indeed, computational visualizations showed that this sphere is eventually entrained into the wake of sphere 1 rather than expelled as in the experiment. We note also that the leading spheres are accelerated more slowly downstream in the computation, probably because of the lack of a viscous drag component. Nevertheless, despite the accrual of these positional errors, the lateral velocities of spheres 1, 2 and 3 do not diverge significantly from the experimental results at later times, with stable errors of 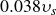 , 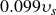 and 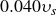 , respectively, at 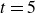 ms. Considering that the separation velocity is the quantity of primary interest here, these errors can be considered sufficiently low for confidence in the equivalence between the simulated and experimental realizations of the separation process. The discrepancy with sphere 4, however, once again highlights the sensitivity of the sphere motions to the exact shock location in impinging configurations.

## 3. Two-sphere survey

The simplest sphere-separation configuration is two equal-sized, initially touching spheres at various alignment angles. The aerodynamics of this configuration have been well characterized in previous studies (Laurence *et al.* 2007; Laurence & Deiterding 2011; Register *et al.* 2020), but the effects of surface contact between the pair have not yet been systematically investigated. We thus begin our investigation by elucidating the physics governing sphere-pair separation for various initial alignment angles. We adopt the convention whereby 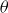 represents the alignment angle between the flow velocity vector and the line connecting the sphere centres, with an angle of 180 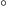 indicating that the secondary (downstream) sphere is directly behind the primary (upstream) sphere. The simulated configurations span initial alignment angles, 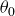 , of 90 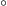 to 180 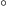 in increments of 7.5 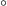 .

**Figure 9.**
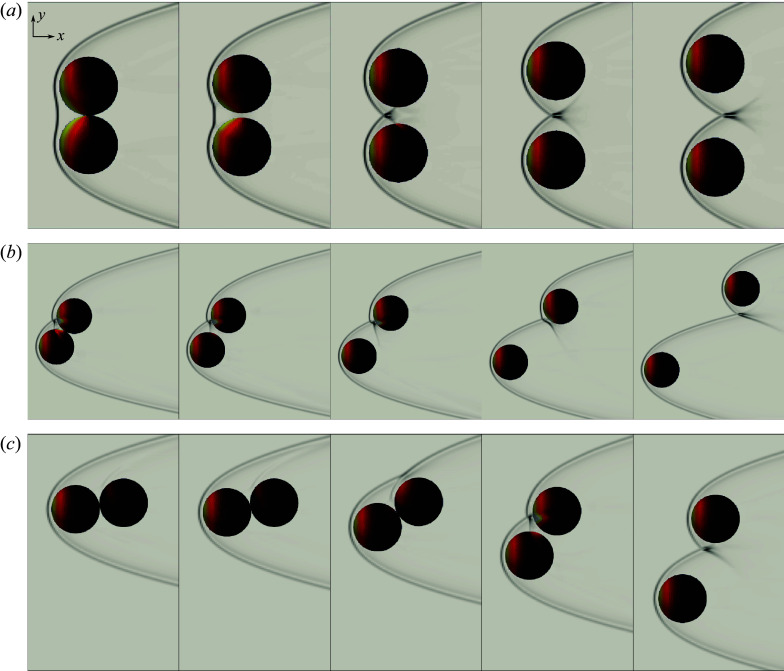
Sphere-pair separation sequences for initial alignment angle of (*a*) 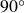 , (*b*) 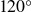 and (*c*) 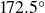 , in increments of 0.61 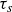 , 1.20 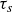 and 2.11 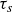 , with colouring by surface pressure and centreline pseudoschlieren.

To acquaint the reader with the basic qualitative aspects of sphere separation, we first present visualization sequences of several representative cases that demonstrate typical sphere-pair behaviours, some of which arise in more populous clusters. Figure 9(*a*) illustrates the dynamics resulting from a well-studied initial arrangement, two bodies positioned at the same streamwise coordinate (i.e. 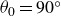 ). The spheres are initially subjected to pressures of similar magnitude to the stagnation-point value on their inboard regions as a result of their common bow shock. The resulting spanwise separation produces a bifurcation of the bow shock and an associated reduction in the extent of the region of elevated inboard pressures; by the fourth frame, they are effectively travelling independently of one another. Rotating the pair to an alignment angle of 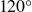 , the separating action of the common bow shock gives way to dynamics dictated by shock impingement on the downstream sphere and the shock-surfing behaviour first noted by Laurence & Deiterding (2011). This significantly augments the streamwise and lateral velocity of the secondary body, but other than the resulting streamwise separation, the ultimate terminal behaviour of the pair relative to their common centre of mass is not all that different from the first case. The same can be said of figure 9(*c*), corresponding to 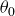 = 172.5 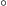 , though the means by which this final lateral separation is achieved is again quite different. In this configuration, the primary body experiences a higher drag than the secondary body, which yields a destabilizing moment. The resulting `rolling’ motion of the spheres is initially imperceptible but accelerates as the spheres’ inclination to the free stream grows, and by the fourth frame has led to a termination of the contact. The secondary body subsequently passes rapidly through the shock of the primary, before the spheres achieve aerodynamic independence in a manner similar to the lower 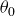 arrangements. However, in contrast to the 120 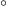 case, the primary sphere accrues more lateral momentum than the secondary, the lateral position of the latter remaining nearly stationary over the simulation; this signifies opposing trends for the motion of the system’s centre of mass for these two cases.

**Figure 10.**
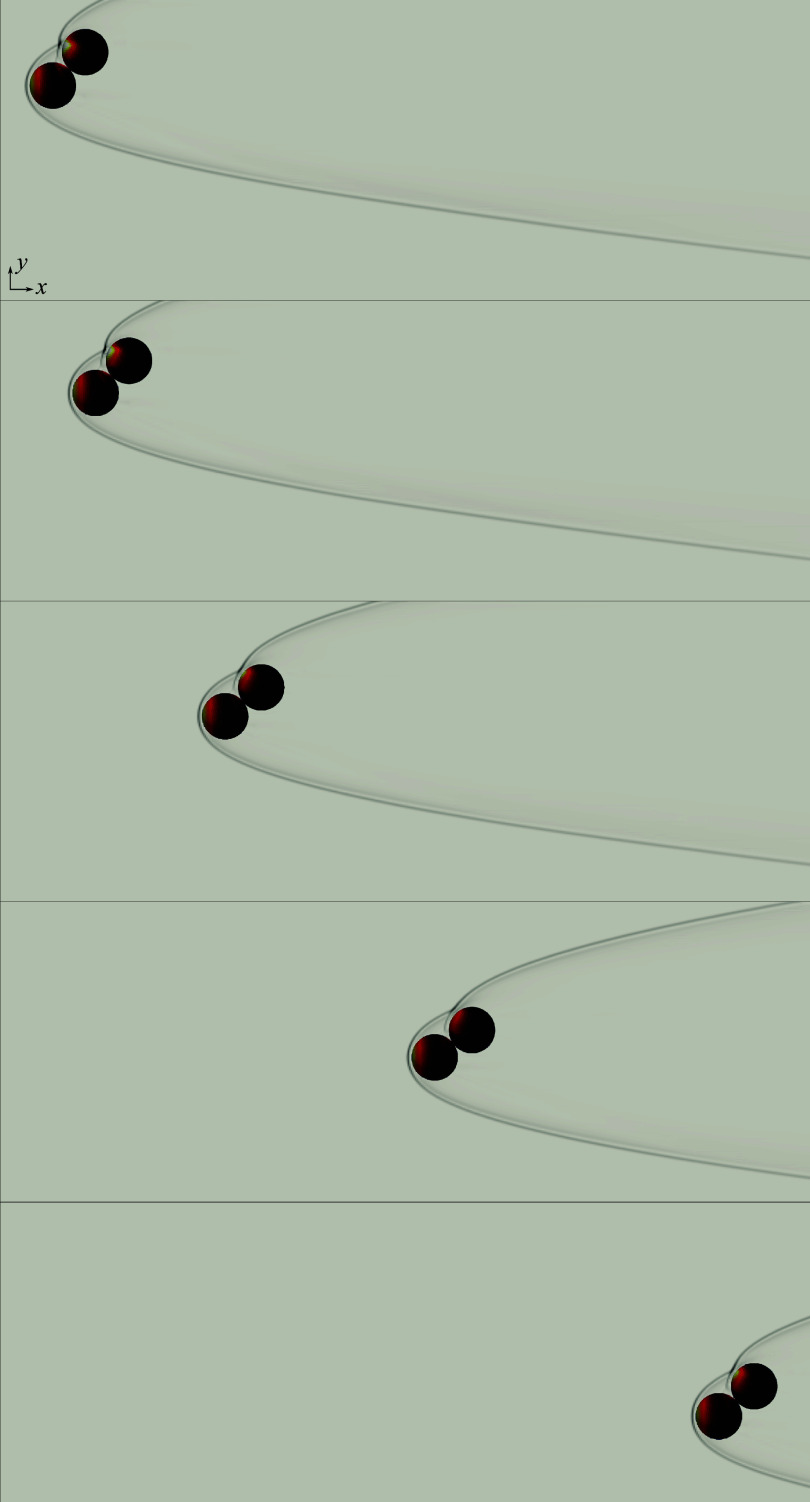
Sphere-pair separation sequence from initial alignment angle of 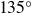 with colouring by surface pressure and centreline pseudoschlieren; panels are shown in increments of 1.83 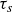 .

For the 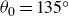 arrangement visualized in figure 10, however, a dramatic change in the spheres’ motions occurs. As in the 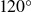 configuration, the secondary sphere is subjected to the bow shock of the primary, but shock impingement now occurs on the upper-half of the body, which promotes prolonged contact between the bodies. Indeed, the bodies travel downstream in tandem, maintaining mechanical contact, and, due to an effective moment provided by the shock impingement, begin to rotate in the 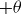 direction about their common centre of mass. The alignment angle of the pair reaches a maximum in the fourth frame and, as evidenced by the reappearance of the high-pressure impingement region in the fifth frame, decreases thereafter, suggesting stable cyclical behaviour. At the same time, the effective angularity of the tandem bodies results in a common lift force (in this case in the negative 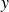 direction) which drives the pair in the lateral direction. In contrast to the previously explored scenarios, this potentially stable lifting behaviour relies on both the aerodynamics and contact mechanics of the constituent bodies and could represent an important mechanism for enhancing the lateral momentum of more populous clusters.

**Figure 11.**
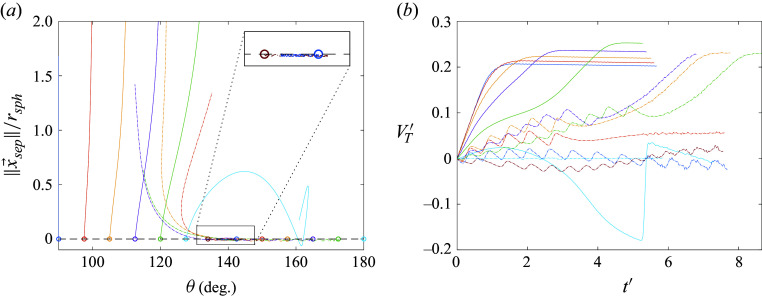
(*a*) Polar trajectory map and (*b*) lateral-velocity histories in two-sphere survey. The inset in (*a*) shows only the 135 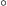 and 142.5 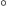 trajectories.

Having established the general trends associated with sphere-pair aerodynamics, we now present in figure 11(*a*) the trajectories of all secondary (downstream) spheres in the polar coordinate system of the primary (where the vertical coordinate represents the normalized edge-to-edge separation of the spheres); the time histories of the lateral sphere velocities from the system’s centre of mass are meanwhile shown in figure 11(*b*). The behaviour near alignment angles of 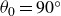 closely conforms to expectations of mutual repulsion examined earlier, with the spheres achieving separation velocities of 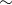 0.2 over their 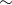 1.7 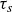 interacting flight duration. As evidenced by the post-release increase in alignment angle for higher 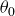 , the transition from mutual repulsion to shock-surfing-induced separation occurs gradually between alignment angles of 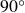 and 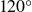 and causes an appreciable enhancement to the lateral spread of the bodies, with terminal 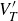 values rising monotonically from 0.2 to 0.25 and the separation time scale extending to 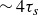 . At 127.5 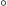 , the secondary sphere exhibits brief surfing before becoming entrained in the wake of and then colliding with the leading sphere (the brief excursion into a negative displacement is caused by deformation of the bodies upon collision). This simulation does not resolve the ultimate trajectories of the spheres, however, leaving open the possibility that a prolonged series of collisions is a sustainable configuration.

**Figure 12.**
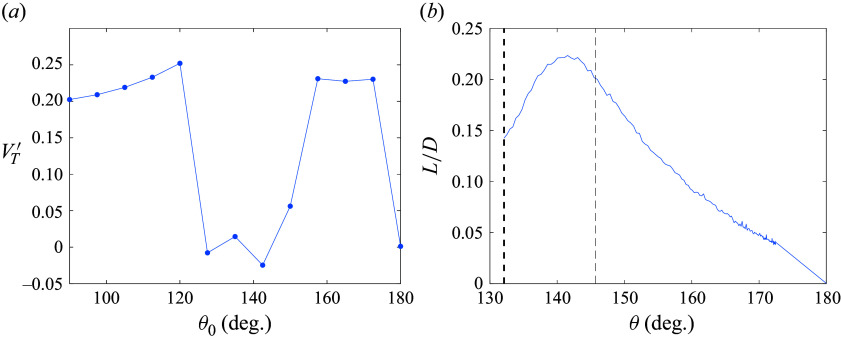
(*a*) Final lateral velocity with initial alignment angle; (*b*) lift-to-drag ratio of contacting sphere pairs, with the 132 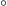 and 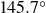 stability limits indicated by dashed lines.

Spheres initially positioned in 135 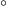 and 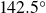 arrangements, in contrast, remain in contact for the duration of the simulations; in both cases, restorative moments drive the angular alignment of the pair about an apparent equilibrium 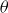 . Indeed, for these two cases, both the surface separation of the spheres and lateral velocity of the system remain centred around zero, with low- and high-frequency residual oscillations representing the bulk rotational oscillations and the elastic mechanical vibrations from surface contact, respectively. Further examination of the forces and moments revealed that this restorative effect acts over a range of angles between approximately 132 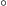 and 145.7 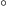 , representing the extent of the stable region for the two-sphere dynamics. As the initial orientation is rotated further towards alignment with the free stream, the trailing sphere tends to exhibit the behaviour exemplified in the lower sequence of figure 9, `rolling’ along the surface of the primary until losing contact and undergoing aerodynamic separation. The 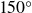 case features a secondary sphere that remains on a persistent shock-surfing trajectory, albeit without much enhancement to its lateral velocity. On the other hand, significant augmentation of the lateral velocity by shock-surfing following the loss of surface contact is observed for cases 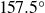 – 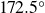 ; in these instances, the angular momentum accrued by the pair endows the secondary sphere with enough lateral momentum to transit the impinging shock. The duration of contact here is somewhat dependent on the initial angle, ranging from 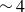 to 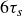 (figure 11 *b*), while the loss of contact in all cases appears to occur near 130 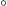 (figure 11 *a*). For an initial alignment angle of 180 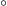 , no discernible change in the pair’s attitude was recorded, suggesting the presence of an equilibrium position (though we expect this to be unstable, given the higher drag of the upstream body in this configuration). Noticeably absent in figure 11(*a*) are secondary spheres that persist in the wake of the primary, which follows from the reduced drag and subsequent collisions that occur in such arrangements. This seems to signify three permitted trajectories – immediate separation, delayed separation following a period of contact, and indefinite contact – with sphere pairs in the former two categories achieving lateral velocities generally between 0.2 and 0.25. The final lateral velocities are shown in the left-hand part of figure 12(*b*), with this bimodal behaviour manifesting itself in the two distinct sets of values.

A particularly important consequence of stable sphere contact is the effective lift resulting from the pair’s asymmetry. Thus, treating the sphere pair as a binary rigid body, the lift-to-drag ratio experienced mutually by the sphere pair is shown in figure 12(*b*). This single curve was created by stitching together data over various angle ranges from multiple simulations (note that the region between 172.5 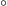 and 180 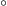 is linearly interpolated). We find a maximum 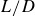 of 0.22 at 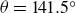 followed by an approximately linear decrease. While the mean 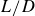 of 0.197 within the stable region is quite low, even for hypersonic aerodynamics (Anderson 2006), unlimited enhancement to the lateral velocity of the system would be theoretically possible. More likely, however, is the occurrence of limited contact, during which significant mutual momentum can still be accumulated. Indeed, for simulations with 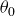 values of 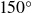 , 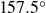 and 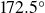 , the terminal centre-of-mass 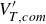 values are 0.42, 0.32 and 0.28, respectively, higher than the individual fragment velocities relative to this centre of mass. Thus, this lifting-pair contact mechanism may constitute an important aspect of equal-sphere separation in more populous settings, as has been confirmed by the experiments of Whalen & Laurence (2021).

## 4. Four-sphere tetrahedral arrangements

### 4.1. General characteristics and influence of initial sphere position

The four-equal-sphere experiments of Whalen & Laurence (2021) illustrated through a small number of representative cases the division of sphere dynamics into distinct primary and secondary regimes: we now conduct a simulation survey of the same tetrahedral geometry to extract a more detailed description of sphere separation from this configuration. Compared with the two-sphere case, where a single angle ( 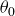 ) was sufficient to prescribe the initial arrangement, we now require two angles. We use the pitch and yaw angle for this purpose, the separation behaviour being degenerate under roll variations. We adopt a convention such that 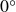 -pitch/ 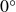 -yaw corresponds to three spheres forming a streamwise-normal plane behind a single leading sphere. The simulated parameter space spans angles of 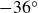 to 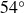 in pitch and 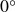 to 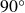 in yaw at intervals of 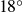 , with several additional tests performed to capture the full extent of separation behaviours.

**Figure 13.**
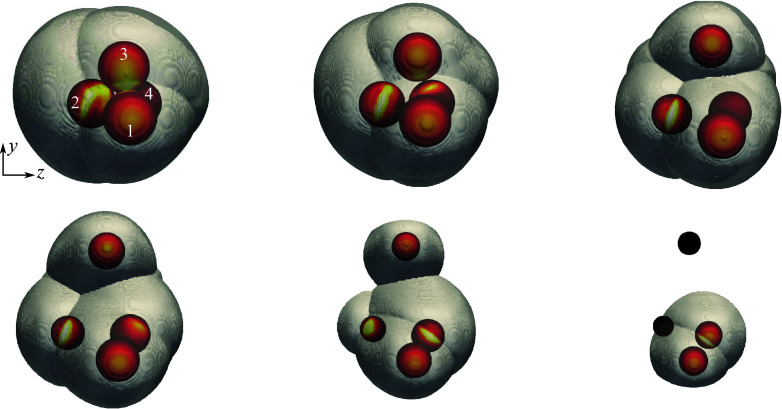
Visualizations of spheres separating from a 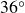 -pitch/ 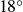 -yaw tetrahedral cluster, with surfaces coloured by pressure and primary shock structure visualized in grey; blacked-out bodies have left the computational domain. Images are in steps of 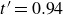 .

**Figure 14.**
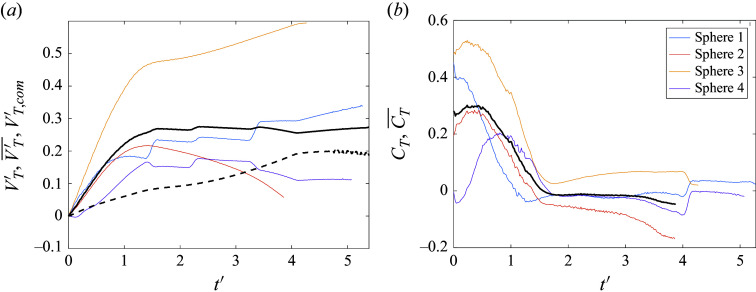
(*a*) Lateral velocities and (*b*) lateral force coefficients for the above four-sphere cluster, coloured by sphere number: solid black 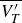 or 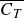 ; dashed black 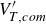 .

First, to highlight some of the governing dynamics of the four-body separation scenario, we review the features of a typical separation sequence. In figure 13, we present a set of snapshots visualizing instantaneous sphere positions coloured by surface pressure and the associated primary shock structures on the first refinement level from a cluster with an initial orientation of 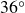 -pitch/ 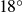 -yaw; corresponding lateral velocities and force coefficients are shown in figure 14. This is the same configuration employed in the grid refinement study of § 2.4, but now explored in additional detail. In the cluster’s initial state, an encompassing bow shock generated primarily by sphere 1 impinges on spheres 2 and 3, while sphere 4 is shielded in sphere 1’s wake. The shock–shock interaction on the inboard side of sphere 3 results in the previously noted region of high surface pressure on both spheres 1 and 3; the relatively large lateral force coefficient on sphere 3 ( 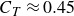 ) promotes immediate repulsion, with this sphere becoming aerodynamically independent from the rest of the cluster by 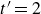 . Sphere 2 resides at a location farther downstream in the cluster and so is initially subjected to shock impingement from sphere 1 that transitions to a swept shock–shock interaction between spheres 1 and 3. The resulting region of higher pressure occurs towards the front of the body, which induces augmented drag and increases the initial impulse in the streamwise direction, while maintaining a highly elevated lateral force coefficient beyond 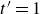 . Sphere 2 subsequently follows an extended shock surfing trajectory before becoming entrained in the bow shock of sphere 1. Sphere 4, on the other hand, is located initially in the wake of sphere 1 and experiences highly reduced drag, which initiates a prolonged series of collisions before it `rolls’ away from sphere 1, as in § 3. The primary separation phase lasts until 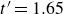 , as inferred from the mean lateral force coefficient, at which point the collective lateral velocity has reached 0.27 and changes little thereafter. The centre-of-mass velocity, in contrast, continues to grow substantially during the secondary phase, reaching a value of 0.2 that is comparable to the collective velocity. While the spheres have not all reached their final aerodynamic state before exiting the computational domain (which is true of several simulations in this survey), the collective quantities – 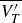 and 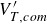 – have essentially plateaued and their final simulated values can be considered generally representative of their terminal states.

**Figure 15.**
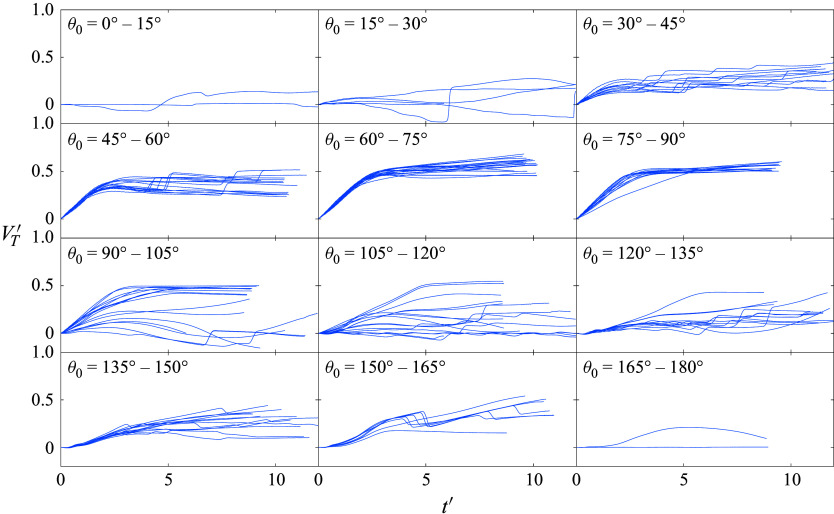
Time-series of lateral velocities of spheres in tetrahedral clusters binned by initial polar angle.

In the two-sphere case, the parameter governing the resultant dynamics is the initial polar alignment angle, and we might expect the initial position of a given sphere within its tetrahedral cluster to also play an important role in the present case. We thus bin all spheres over the survey into groups of 15 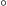 width by initial polar angle within the cluster and compute the magnitude of the lateral velocity of each body relative to its cluster’s centre of mass. In figure 15, the stacked time series of grouped lateral velocities show a clear dependence of trajectory on initial polar angle, 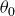 . First, spheres positioned towards the front of the formation ( 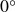 – 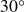 ) tend to experience mild separation velocities, likely because the downstream spheres are largely incapable of influencing their dynamics. At intermediate forward positions ( 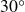 – 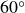 ), the separation characteristics seem dominated by collisions with other spheres, which manifest themselves as discontinuous jumps in 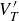 . This category is mainly occupied by the leader of a sphere pair repelled in tandem (e.g. spheres 1 and 4 in figure 13), and the enhancement in 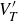 through the two-sphere subcluster interaction can reach values of 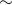 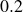 , which is consistent with the results of § 3. Spheres located slightly upstream of the flank ( 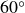 – 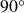 ), in contrast, are consistently subject to immediate expulsion or shock surfing, reaching relatively uniform and high lateral velocities close to 0.7. Polar angles above 90 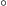 mark a transition from expulsion to entrainment trajectories, although the notable variation in separation velocities for 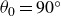 – 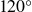 indicates some level of dependence on geometry-specific properties. Just as bins 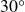 – 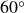 constitute in many cases the upstream (primary) bodies in a sphere pair, the spheres in bins 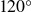 – 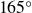 represent their downstream (secondary) counterparts; these trajectories, too, are marked by collisions and a delayed increase in lateral velocity typical of the `rolling’ interactions. Finally, in the rear of the formation, rotational symmetry limits the lateral separation velocities to modest values.

**Figure 16.**
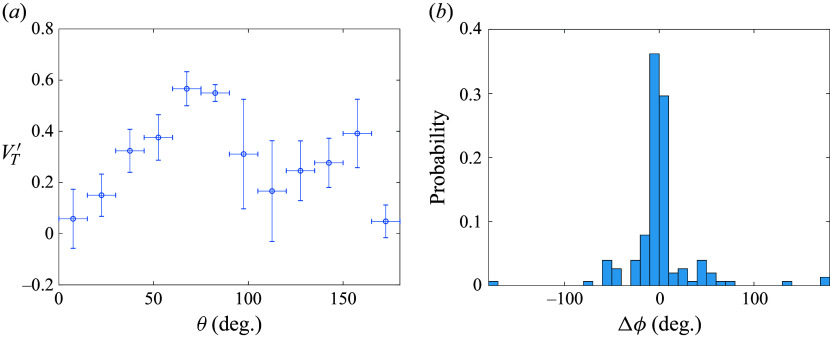
Ensemble statistics of spheres in tetrahedral clusters examining (*a*) terminal lateral velocity binned by initial polar angle and (*b*) difference between final and initial azimuthal angle.

Using these binned trajectories, we can now examine how well a sphere’s initial polar angle within the cluster predicts its final separation velocity. Defining the final velocity as that attained when either the sphere reaches the limits of the computational domain or the simulation ends, we present the mean lateral velocity of each binned group, along with error bars denoting one standard deviation, in figure 16(*a*). The lateral velocity increases nearly linearly over initial polar angles from 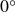 to 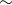 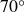 , the latter marking the angle at which extended shock surfing is most probable. The brief plateau between 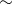 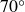 and 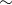 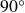 is consistent with the high sensitivity of shock surfing to initial positioning and represents the highest degree of expulsion observed in the survey. A substantial reduction in separation velocity occurs thereafter as a result of the increasing number of entrainment events, although a wide spread in the data is apparent. Above 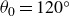 , we again observe a roughly linear rise in the separation velocity; these bins represent spheres that are initially shielded from the free stream flow by an upstream body and remain in the wake of that body until a nominal two-sphere interaction can commence. Close to 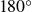 , tetrahedral symmetry appears to keep the separation velocity low, but the sample size is too small to draw any reliable conclusions.

We have just seen that the initial polar angle determines to a large extent the magnitude of the final separation velocity for a given fragment, but the direction of this velocity would also be important for modelling such separation events. Existing models of the aerodynamic separation of fragmenting bodies (the pancake model of Hills & Goda (1993), for example) assume pure radial expansion of the free-flying bodies, but, to the authors’ knowledge, this assumption has not been properly verified. In figure 16(*b*), we thus show the total change in azimuthal angle, 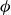 , for each sphere in the survey, referenced to the respective cluster centre of mass. We see that the majority of spheres experience only a limited change in their azimuthal positions: 75 % remain with 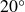 of their initial values, while a handful of entrained bodies pass through the wake of a primary sphere to achieve 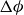 values of close to 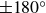 . A moderate number of bodies stray farther from their initial azimuthal positions, though tetrahedral symmetries are likely a contributing factor here, as such cases tend to occur with pairs of trailing spheres near initial yaw angles of 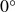 . Overall, despite some variation in azimuthal angle, we find that the preponderance of bodies expand nearly laterally from their initial positions, justifying to some extent the use of this assumption in simplified aerodynamic modelling of fragmentation events with equal-sized bodies.

### 4.2. Effects of cluster orientation on bulk behaviour

Now that we have established some broad conclusions regarding the separation behaviour of spheres based only on their initial positioning within the cluster, we turn to the bulk separation characteristics of the tetrahedral clusters, particularly as determined by their initial orientations. The pitch and yaw values used thus far are convenient parameters for characterizing the cluster orientations, but we do not necessarily expect them to exhibit any particular physical relevance to the separation behaviour of a given cluster. We thus introduce a set of reduced parameters that are functionally independent of the specific configuration considered and better capture the geometric features that one might expect to be significant for the resulting aerodynamics. First, we define a bluntness parameter, 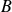 , which effectively describes the degree to which the primary bow shock is generated by multiple spheres, thus creating a region of high pressure between the bodies and promoting immediate separation. For a given tetrahedron orientation, 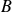 is determined by computing the unit normals for all external faces (i.e. planes through any three sphere centroids), 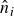 , and finding the maximum projection onto the upstream direction:
(4.1)

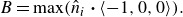

The geometry of a tetrahedron limits the bluntness index to (1/3, 1), where larger values indicate multiple spheres facing into the flow, and thus an expected higher proclivity for immediate repulsion, and the smallest values denote a single leading sphere.

The second parameter is the asymmetry index, which represents the lateral mass offset between the aerodynamically relevant bodies and the system’s centre of mass, i.e. this parameter takes a larger value when upstream (i.e. shock-generating) bodies are located away from the centreline. To compute this quantity, we determine the initial lateral distance of each sphere from the centre of mass and assign a summation weight based on its streamwise position; a quadratic weighting is utilized upstream of the centre of mass, while downstream spheres are assigned a value of zero. The set of weights is normalized to unity, and the two-norm of the summation is scaled by the radius of the virtual circumscribing sphere, i.e.
(4.2)

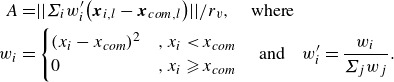

**Figure 17.**
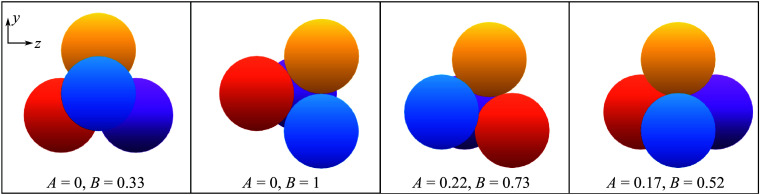
View from directly upstream of sample clusters labelled according to geometric parametrization.

Here, the 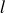 subscript refers to the lateral component of the position vector and 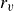 is the virtual sphere radius. The asymmetry index takes values between 0 and 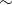 0.32 for a tetrahedron, where the aerodynamic offset from the primary axis is greatest at larger values. To provide the reader with an intuition for the appearance of clusters at certain points in 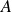 – 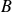 space, in figure 17 we present several agglomerations that exhibit slender, blunt, symmetric, asymmetric and mixed attributes. The locus of points in the 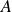 – 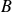 plane is highly dependent on the cluster geometry and appears much more irregular in form than in the pitch–yaw space explored for survey purposes. Furthermore, we note that roll-degenerate states are mapped to the same coordinates in 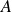 – 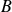 space, as desired.

**Figure 18.**
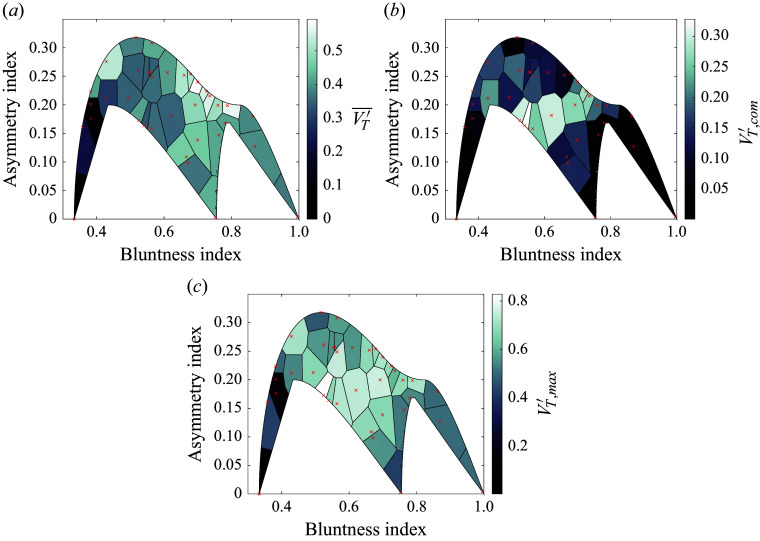
Maps of (*a*) collective separation velocity, (*b*) lateral centre-of-mass velocity and (*c*) maximum lateral velocity under the reduced parametrization for tetrahedral clusters.

**Figure 19.**
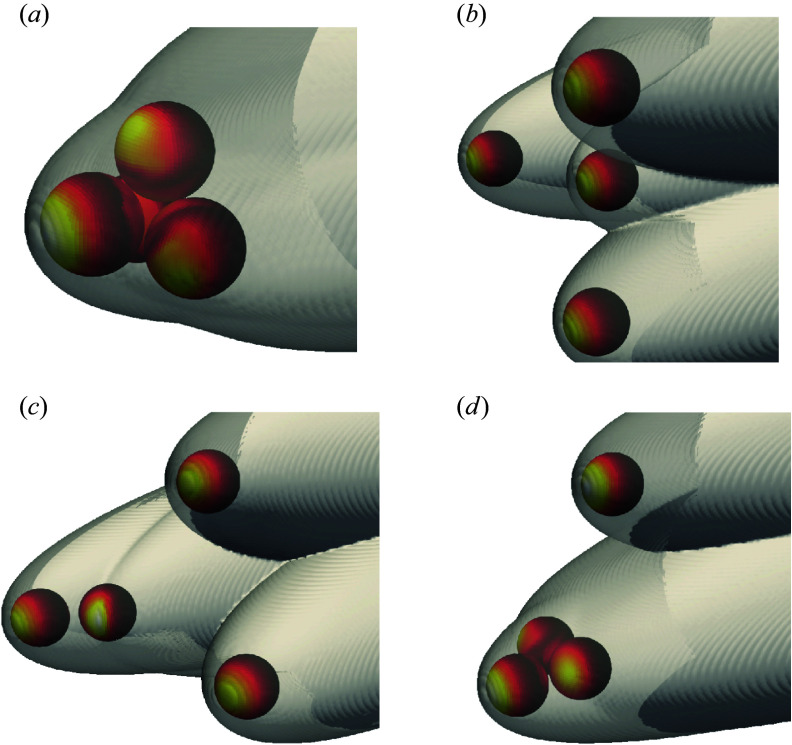
Instantaneous visualizations of tetrahedral cases with asymmetry–bluntness indices of (*a*) 0–0.33, (*b*) 0–1, (*c*) 0.22–0.73 and (*d*) 0.17–0.52, demonstrating the dependence of separation characteristics on cluster geometry/attitude. The selection of cluster attitudes is identical to that in figure 17.

In figure 18, we present maps of the surveyed cluster separation properties under the reduced parametrization. In each map, the data points corresponding to the simulations are shown as red crosses, with the vertex-centred tessellations shaded according to the relevant quantity (the collective separation velocity of the cluster, 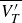 , the lateral velocity of its centre of mass, 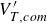 , and the maximum lateral velocity, 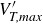 ); the map boundary was derived from a random sampling of cluster orientations. Illustrative visualizations for several cases, taken at the same time step in each simulation, are shown in figure 19. The map of 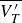 in figure 18(*a*) demonstrates a clear correlation of the collective separation velocity with the bluntness index. Near 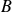 values of 1/3 (i.e. more streamlined cases), the cluster approaches separation velocities of zero. Extrapolating the results of § 3 might lead one to expect a lower limit of 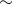 0.2, but certain arrangements possess symmetries that render them somewhat resistant to separation. In figure 19(*a*), for example, which shows an 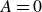 , 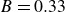 cluster, increased outboard pressures on the rear spheres due to shock impingement are balanced by contact forces, allowing the cluster to translate downstream without any perceptible disruption. This specific behaviour may be particular to the idealized tetrahedron–sphere configuration and not representative of actual entry events, but one would nevertheless expect relatively low separation velocities to result from such streamlined configurations. Towards higher values of 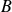 , 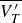 increases to 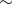 0.5, which tends to occur when all spheres are immediately repelled. While one might expect little dependence of the collective separation velocity on the asymmetry index, 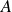 , we see that a larger aerodynamic offset can enhance separation compared with initially blunter clusters with high symmetry. In the visualization of figure 19(*b*) ( 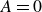 , 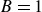 ), the presence of one trailing sphere along the primary axis reduces the overall lateral velocity of the cluster ( 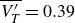 ), whereas all spheres (including a primary/secondary pair) are repelled from the centre of mass in the example shown in figure 19(*c*) ( 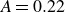 , 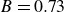 , 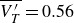 ). To estimate a characteristic 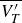 for tetrahedral clusters, we weight each simulation by its normalized cell area in figure 18(*a*), finding a value of 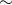 0.35; this represents a marked increase from the average sphere-pair value ( 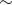 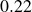 , considering variations in the azimuthal angle).

From figure 18(*b*), the cluster centre of mass can deviate significantly from the initial primary axis. The area-weighted mean of the tessellations yields a value of 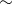 0.13, large enough to meaningfully alter the trajectory and location of energy deposition of a body entering the atmosphere. Surprisingly, the correlation with the asymmetry index is not so clear here. Highly symmetrical cases (such as shown in figures 19 *a* and 19 *b*) do tend towards a laterally stationary centre of mass, but the most severe deviations of centre of mass occur for only moderately asymmetric agglomerations. For instance, figure 19(*d*) ( 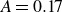 , 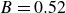 ) shows a quasistable subcluster of three spheres that skews the centre of mass sharply away from its initial position. For higher asymmetry values, primary/secondary sphere pairs can generate transient lifting configurations before eventually separating, potentially enhancing the offset velocity: such a pair is seen in figure 19(*c*), but this pair’s lateral momentum is almost completely offset by two bodies initially expelled from the cluster.

Finally, with a focus on potential outlying fragments in a strewn field, in figure 18(*c*) we examine the maximum velocity amongst the expelled bodies within each cluster. Interestingly, the maximum velocities appear to be roughly consistent with the sum of the collective lateral velocities and centre-of-mass offset, although there is no clear reason why this should be the case. The maximum lateral velocity of the survey (0.82) occurs in the lifting triplet of figure 19(*d*) and is almost 2.5 times greater than the mean 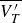 . The majority of expelled spheres with 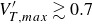 arise due to shock surfing and/or paired travel; the area-weighted average of 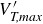 is 0.54.

## 5. Thirteen-sphere arrangements

### 5.1. General characteristics

In all clusters examined thus far, every sphere has been partly exposed to the external flow field. For close-packed clusters with 13 or more spheres, however, at least one body must be located wholly on the interior, which may significantly alter the separation mechanics. Therefore, we now advance to a population of 13 spheres – the next highest population after four with a quasispherical face-centred cubic structure – and simulate the separation process for a large selection of initial orientations. The base geometry is established by constructing a face-centred-cubic lattice outwards from a single central body, with the orientation of the lattice chosen such that a group of seven spheres forms a honeycomb-like pattern along the 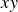 -plane with one vertex pointed directly upstream. We then vary the pitch and yaw of the cluster about the central body according to two criteria: one half of the survey was selected from equally spaced points in the same reduced pitch–yaw space employed for the four-sphere computations, while the remaining simulation attitudes were randomly sampled from a uniform distribution. Figure 4 gives a schematic of the base 13-sphere-cluster configuration and geometric definitions. As in the four-sphere case, spheres sometimes exited the computational domain before the simulation had concluded, which prevents precise characterization of all terminal trajectories; however, while these bodies were occasionally still undergoing aerodynamic interactions, the majority of the cluster’s overall lateral momentum has already been attained, so any increases gained farther downstream are expected to be marginal in most cases.

**Figure 20.**
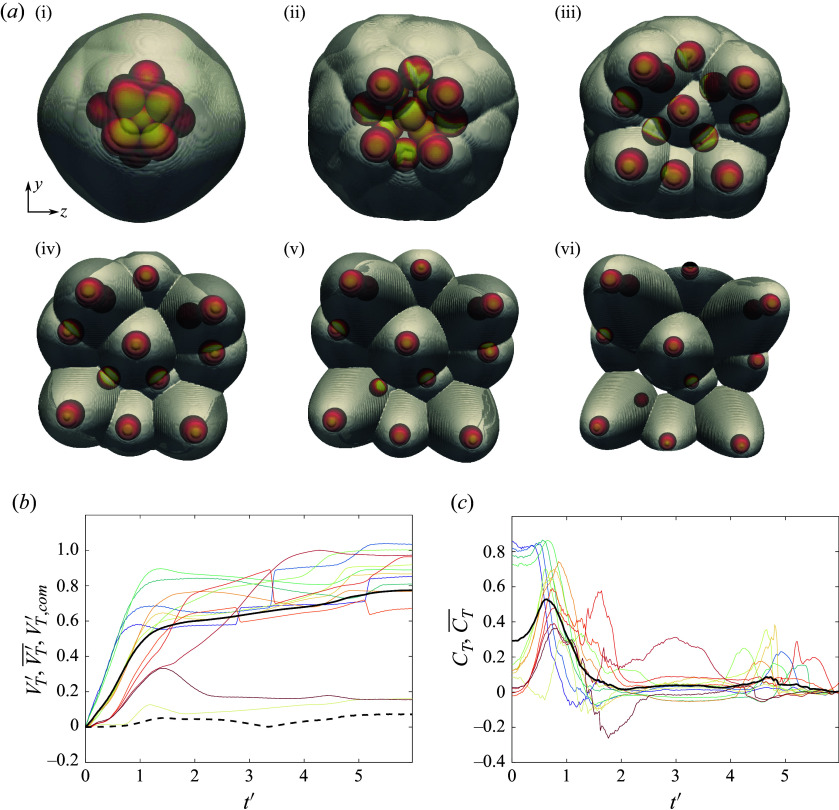
(*a*) Downstream-projected trajectories of 13-body cluster at 136.8 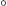 pitch and 141.1 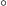 yaw, with spheres coloured by surface pressure and translucent bow shocks extracted from pseudoschlieren; images shown in increments of 0.83 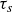 . (*b*) Time-varying separation velocities of individual bodies together with 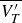 in solid black and 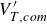 in dashed black; (*c*) individual lateral force coefficients with mean value ( 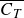 ) in solid black.

First, to elucidate some of the separation behaviours characteristic of 13-sphere clusters, we present the results of two selected simulations exhibiting somewhat opposing characteristics. In figure 20, we analyse the trajectories and forces of spheres originating from a cluster rotated 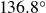 in pitch and 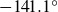 in yaw (additional figures in which these results are split into two sphere groups for easier viewing are provided in the Supplementary material). From the first flow visualization (figure 20 *a* i), we see that this configuration is highly blunted, with the most upstream four spheres producing a plane lying nearly normal to the primary axis. An additional five spheres are partly exposed to the free stream, forming a plane behind the foremost layer, while the remaining four are completely shielded farther downstream. At the moment of sphere release, a common bow shock encompasses the entire cluster, with little evidence of shock impingement, and high inboard pressures occur on the surfaces of the upstream layer. The central body is likewise subjected to the high-pressure streamlines, but, owing to the cluster symmetry, the resulting forces are predominately directed downstream. As the cluster begins to separate, the upstream spheres are repelled under roughly constant lateral forces ( 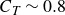 in figure 20 *c*), separating enough to allow the shocked inflow to influence the motions of the first layer of initially shielded bodies, whose lateral force coefficients rise at 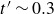 .

By 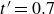 , the internal mass flux has ostensibly increased enough for the cluster to ingest the common bow shock, leading to its collapse into an intersecting set of individual bow shocks by frame 2 (figure 20 *a* ii) and the subsequent loss of lateral force on the upstream layer. The ingested shock structure yields a series of impingements and reflections that propagate to all downstream bodies in the cluster, evident in the 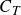 maxima (and the collective 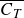 maximum) near 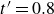 . Further division into independent shock systems progresses with increasing sphere spacing, leading to a diminishing mean lateral force coefficient; the minimum at 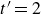 signifies the termination of the primary separation phase. At this point, the collective lateral velocity has reached a value of 0.60 (figure 20 *b*), with the most expelled spheres having originated from the upstream layer. The secondary phase begins between frames 3 and 4 (figure 20 *a* iii and *a* iv); this is marked by a more limited set of impinging shock interactions and the commencement of entrainment–collision sequences, manifest in the lateral velocity profiles of several bodies. These interactions proceed according to kinematics in the reference frame of the leading body of a given subcluster. Indeed, the computational visualizations of frames 5 and 6 (figure 20 *a* v and *a* vi) reinforce the notion of independent subclusters, each bounded by its own leader’s bow shock. The collective lateral velocity continues to rise during this secondary phase to a terminal value of 0.77, a 28 % increase from the end of primary separation. Meanwhile, the lateral centre-of-mass velocity reaches a value of 0.07, i.e. is only slightly offset from the primary axis.

**Figure 21.**
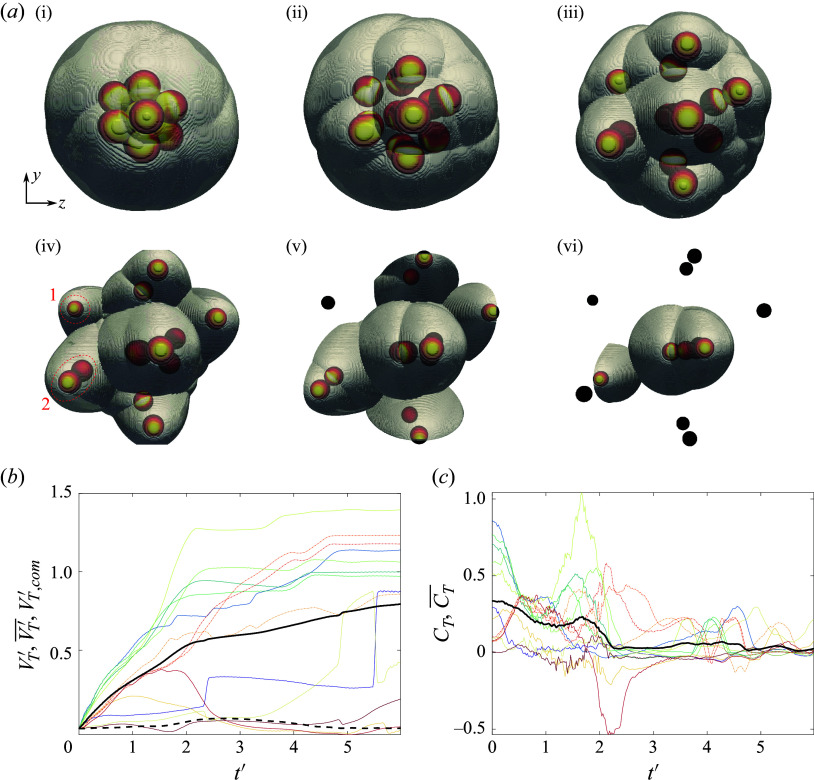
(*a*) Downstream-projected trajectories of 13-body cluster at 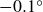 pitch and 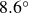 yaw, with spheres coloured by surface pressure and translucent bow shocks extracted from pseudoschlieren; images shown in increments of 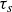 . (*b*) Time-varying separation velocities of individual bodies together with 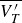 in solid black and 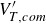 in dashed black; (*c*) individual lateral force coefficients with mean value ( 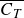 ) in solid black.

For the second representative simulation, we consider a slenderer geometry oriented at 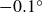 -pitch/ 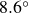 -yaw to the free stream and displayed in snapshots in figure 21(*a*). Here, a lone upstream sphere generates a bow shock that impinges on six downstream bodies, leaving an additional six bodies completely shielded from severe pressure loading. In contrast to the narrow bands of elevated pressure observed in more isolated shock-impingement situations, broad regions of high pressure are apparent in the first frame (figure 21 *a* i), resulting from the subsonic flow following a ring of terminal normal shocks. At the start of the simulation, five of the exposed bodies exhibit large lateral force coefficients – 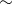 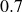 on average – while the remainder are subjected to more modest aerodynamic repulsion. Inspection of the lateral velocity and force profiles reveals an apparent discrepancy in the motions of three bodies (with relevant curves plotted dash–dotted): despite experiencing pressure loading substantially less than the mean through 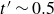 , these spheres are expelled with speeds comparable to the collective lateral velocity, indicating that mechanical contact is a significant factor for these three bodies. As in the previous case considered, the forces on the windward spheres diminish rapidly, reverting to the cluster mean by the frame 2 ( 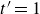 ). The subsequent dynamics are more complex, with further shock interactions resulting in second peaks in 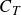 near 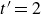 in certain cases. Similar peaks are observed for some of the leeward spheres (in such cases representing a larger fraction of the overall repulsion), while other bodies remain in the aerodynamic shadow of the leading body for extended periods. After its secondary maximum near 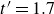 , the mean lateral force coefficient vanishes at 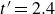 , at which point the collective lateral velocity reaches 0.57 and the secondary separation phase begins. During this phase, a lifting pair (labelled 2 in figure 21 *a* iv) represents the next-most ejected bodies of the cluster after the sphere positioned within a triple-shock intersection to the upper left of the cluster (labelled 1 in figure 21 *a* iv). Meanwhile, the cluster core persists to the end of the simulation and remains to be disrupted by collisions. As a result of this ongoing interaction, the collective lateral velocity has not yet plateaued and is expected to exceed its final simulated value of 0.79, the highest recorded in the present survey.

**Figure 22.**
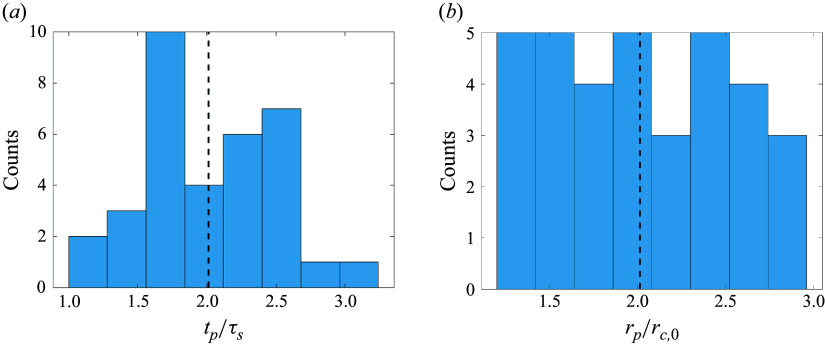
(*a*) Normalized primary separation duration and (*b*) resulting normalized cluster radius for 13-sphere simulation survey.

In both 13-sphere cases just examined, we have witnessed the importance of the primary phase for the overall cluster separation dynamics. We thus now investigate the duration of this phase and spatial expansion caused by mutual fragment repulsion during it. The concept of fragment decoupling time scales and radii in the context of the binary separation problem was introduced by Passey & Melosh (1980). To estimate the duration of primary separation in the present multibody case, we search for minima in the mean lateral force coefficient, 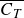 , and select either the earliest minimum for which the local value of 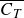 does not exceed 10 % of its maximum or a point of zero lateral force, whichever comes first. We then compute the radius of sphere circumscribing all bodies at the selected time, from which we can estimate the extent of spatial expansion corresponding to aerodynamic decoupling. Histograms of the normalized primary separation time scale, 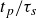 , and normalized decoupling radius, 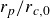 , are presented in figure 22. The 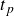 values do not follow any familiar distribution; rather, we observe a prominent maximum at 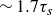 and, with the exception of this peak, a monotonic increase in frequency from 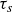 to 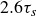 , followed by a rapid drop-off for longer-duration interactions. While the cause of the 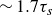 peak is uncertain, it may follow from geometric idiosyncrasies in the construction of the close-packed clusters. The mean primary time scale of 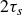 lies between the estimated values for the 11- and 36-sphere experiments of Whalen & Laurence (2021) ( 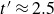 and 1.8, respectively). The decoupling radius, conversely, shows a nearly uniform frequency distribution between 1.4 and 2.9 times the initial cluster radius, with a slight negative dependency with increasing 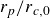 in this range. Furthermore, the mean 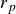 of 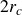 is comparable to the estimated decoupling radius ratio for a 36-sphere cluster in Whalen & Laurence (2021), potentially suggesting a relative insensitivity of this parameter to cluster population. This value is also consistent with the recommendation of Artemieva & Pierazzo (2009) for debris-cloud separation. Interestingly, we find no evidence of a maximum in this distribution, despite the prominence of the 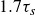 peak in the primary time scale distribution.

### 5.2. Influence of initial sphere position

**Figure 23.**
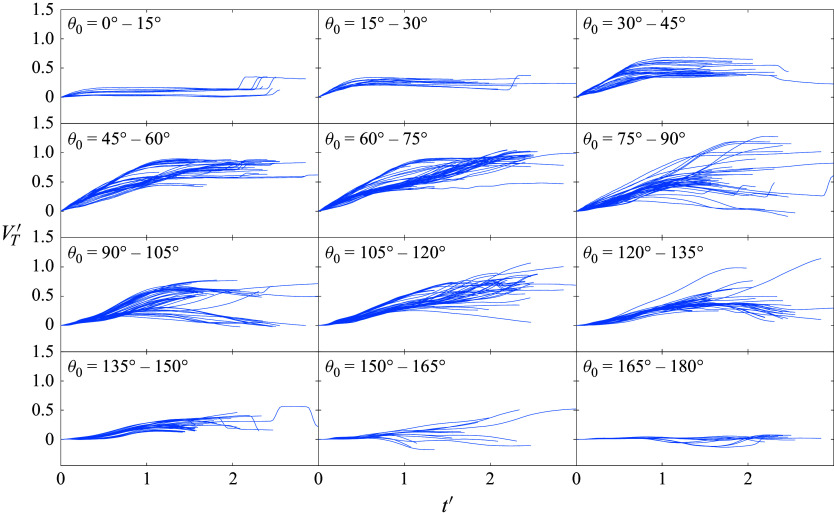
Lateral velocity time series for 13-sphere clusters during the primary separation phase, binned by initial polar angle .

From the visualizations of figures 20 and 21, the intricate system of shocks driving the primary separation of 13-sphere clusters appears to be very much subject to the finer details of relative sphere motions. Nevertheless, we found in § 4 that the terminal lateral velocities in the four-sphere clusters correlated strongly with the initial polar angle, and we thus wish to assess the extent to which a similar relationship exists for more populous clusters. Ignoring for the moment the kinematics of the central sphere in each cluster, for which the polar angle is undefined, we bin all other spheres in the survey by initial polar angle and calculate their transverse velocities through the termination of primary separation; the resulting binned velocity curves are plotted in figure 23. In the 0 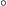 –15 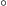 group, the limited radial range inhibits the generation of high-inboard-pressure regions and yields spheres that are forced only gently in the lateral direction and become aerodynamically independent shortly after release. This bin is composed largely of upstream bodies that shape later secondary interactions and, as such, tend to experience collisions near the phase transition point. Still relatively far upstream, the spheres of the 15 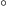 –30 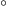 bin are also subjected to relatively limited repulsion durations, but their increased radial positioning endows them with additional lateral momentum. This trend continues for spheres in the 30 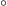 –45 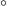 bin, which are exposed to greater forces over a longer duration, resulting in characteristic lateral velocities of 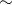 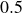 .

The 45 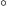 –60 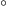 bin exhibits an increased tendency towards bimodality in its lateral velocity profiles: both sudden and delayed repulsion are evident, although the bodies tend to approach similar final velocities. For reference, this and the previous group contain the upstream spheres in the first example simulation shown in figure 20. The 60 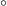 –75 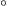 bin shows superficially similar profiles, but more weighted towards trajectories with delayed repulsion, consistent with shock-surfing behaviour; these bodies both reach generally higher final velocities and exhibit inflection points indicative of highly unsteady aerodynamics. As this bin is composed of external shock-receiving bodies (such as those in the second example simulation in figure 21), the applied forces are highly sensitive to the motions of the upstream, shock-generating spheres. Bin 75 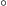 –90 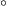 contains highly variable trajectories that encompass a range of terminal behaviours including shock surfing, wake entrainment, and aerodynamic independence. Advancing to the leeward side of the cluster ( 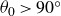 ) marks a transition to dynamics dominated by internal shock impingement following the collapse of the initial collective bow shock. Indeed, the delayed onset of repulsion over 90 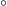 –105 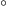 is indicative of this particular phenomenon, although we see that both aerodynamic independence (high final 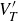 ) and entrainment (low final 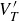 ) remain well-represented as terminal behaviours in this bin. Lateral velocities for 105 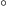 –120 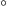 appear to increase throughout the primary phase, suggesting delayed but extended shock-surfing behaviour, with entrained trajectories relatively scarce. For the 120 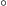 –135 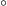 bin, the trajectories show a similar tendency until 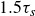 , at which point the lateral velocities seem to diminish considerably, indicative of weak shock surfing leading ultimately to entrainment near the axis of the cluster centre of mass. Finally, at the rear of the formation (135 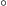 –180 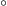 ), aerodynamically shielded spheres near the system’s primary axis experience only limited repulsion. Overall, despite some variability near 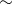 90 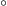 , we conclude that the initial polar angle of a sphere within the cluster again provides a good prediction of its separation tendency and ultimate lateral velocity.

**Figure 24.**
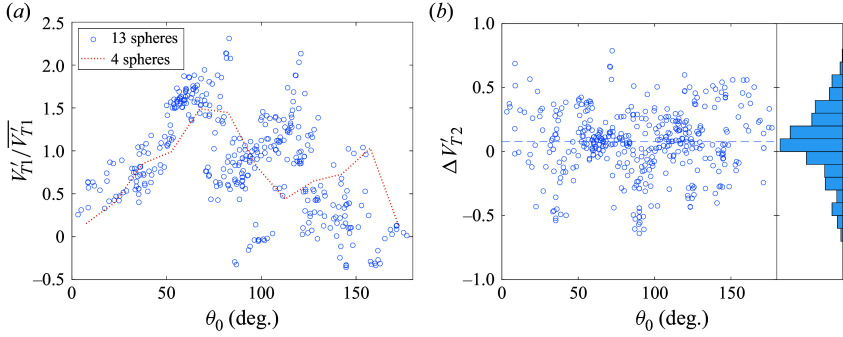
(*a*) Final primary-phase lateral velocity versus initial polar angle, normalized by survey mean and compared with four-sphere survey binned averages; (*b*) lateral velocity accrued during the secondary separation phase, with the mean value denoted by a dashed line and the corresponding frequency distribution on the right-hand side.

We now compile the lateral velocities attained at the end of the primary separation phase, 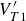 , for each sphere over the survey and, in figure 24(*a*), show their relationship with the initial polar angle. For comparative purposes, we scale these values by the mean lateral velocity of the entire survey (0.55) and include the mean of each 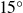 bin from the four-sphere survey of § 4. We see that the general structure of the earlier velocity/polar-angle correlation is retained, despite the vast differences in the detailed separation characteristics of the two populations. Both the four- and 13-sphere clusters exhibit an initial linear regime wherein the degree of expulsion is approximately proportional to the initial polar angle of the given body. Both datasets appear to reach maxima near 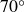 , corresponding to those spheres subjected to prolonged shock impingement, but the trends diverge somewhat farther downstream. For 13 spheres, a steep drop in 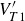 occurs at 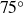 , whereas the four-sphere clusters exhibit a more gradual decline between 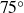 and 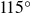 ; this results in an effective offset of the downstream (rising) linear regime between the two populations. Such discrepancies point to differences in the underlying physics: the downstream linear rise for four bodies ( 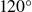 – 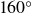 ) results from the tendency of such spheres to form lifting-pair arrangements, whereas, for 13 spheres, the 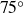 – 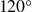 rise represents the influence of internal shock impingements following collapse of the initial common bow shock. Even farther downstream, aerodynamic shielding suppresses separation in both surveys.

While the sphere behaviour follows a relatively consistent trend with initial polar angle during the primary separation phase, the secondary phase proves a considerably less-ordered process. This is demonstrated in figure 24(*b*), in which we effectively repeat the above analysis for the secondary phase. We see that the change in lateral velocity due to secondary separation, 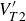 , demonstrates no significant correlation with polar angle. The only discernible features are the lack of radially inward velocity changes near 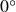 and 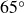 , reflecting the on-axis positioning and severe expulsion, respectively, of spheres in the vicinity of these polar angles. Furthermore, the frequency distribution shown to the right-hand side assumes an approximately Gaussian form, indicating a primarily stochastic nature to this interaction regime. The mean value of 0.077 is consistent with the reduced impact of secondary separation with larger cluster populations, but the comparatively large standard deviation (0.25) indicates that any sphere may undergo significant trajectory modifications after primary separation concludes.

### 5.3. Effects of cluster orientation on bulk behaviour

**Figure 25.**
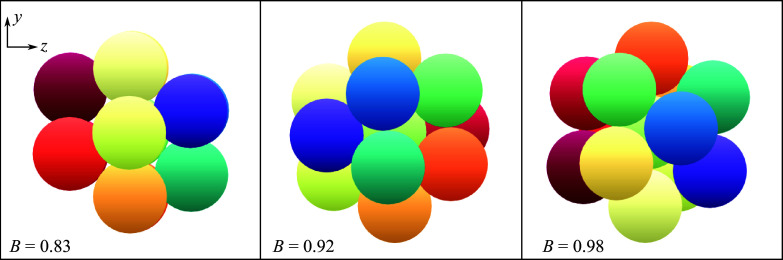
Sample 13-sphere clusters (viewed from upstream) illustrating various bluntness indices.

Having examined the individual sphere motions, particularly as influenced by their initial locations within the agglomeration, we again turn to bulk cluster quantities and examine their dependence on the cluster’s geometrical parameters. For the 13-sphere configuration, the asymmetry index of (4.2) reduces to a negligible value of order 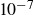 for all orientations (c.f. the maximum of 0.32 for four spheres) and appears to exert no influence over the cluster dynamics. Instead, we focus exclusively on the bluntness index, which has been slightly redefined to break a degeneracy between faces composed of three and four spheres as
(5.1)

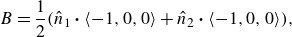

where unit-normals 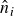 have been sorted by the value of their 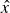 -components. This index, now based on the orientation of the two most-exposed triangulated faces, varies between values of 0.8 and 1.0, with the sample clusters of figure 25 illustrating the variation in appearance over a range of 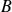 values.

**Figure 26.**
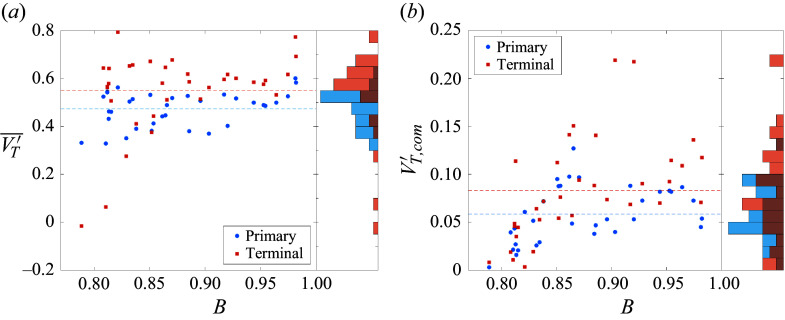
(*a*) Collective lateral velocity and (*b*) centre-of-mass offset with bluntness index after primary and secondary phases for 13-sphere clusters; mean values are indicated by dashed lines and frequency distributions of all quantities are shown to the right-hand side of each plot.

In figure 26(*a*), we present the collective lateral velocity, divided into its postprimary-phase and terminal values, as a function of the bluntness index. The mean primary and terminal 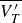 values of 0.474 and 0.551 are indicated by dashed lines. In general, we observe significant scatter about these mean values for lower 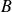 indices, while in each case 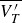 tends to lie above the respective mean for 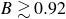 , resulting in a weakly positive trend for 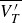 with increasing 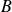 . This behaviour would support the notion that an ingested bow shock can more effectively repel initially shielded bodies in a blunter configuration. The increase in mean 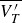 of 16 % during the secondary phase represents a modest overall augmentation, but this is not uniformly reflected across the survey. While most agglomerations do experience enhanced separation after the primary phase, a handful (near 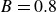 ) approach near-zero values of 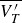 due to inwardly directed lifting pairs (and triplets) and aided by rotational symmetries in the affected configurations. While the permanent stability of these contracting clusters is doubtful, especially considering the eventual energetic collisions that may occur, they represent interesting examples of resistance to separation when oriented bodies are involved, such as may occur in realistic fragmentation events. Projecting these data along the figure abscissa, the resulting frequency histograms assume non-standard forms. The primary-phase velocities peak strongly at 0.52, whereas the terminal velocities show a strong skewness towards values above 0.55. Overall, comparing with the four-sphere results, we conclude that the effects of cluster orientation on the collective lateral velocity become weaker as we move to larger populations. Given that even the present 13-sphere population is relatively small, we might expect such orientation effects to become entirely negligible for more populous regular clusters.

While the centre-of-mass velocity shift is of lesser overall importance for meteor disruption than the collective lateral velocity, it can still hold significance for the precise location of a damage ellipse. In figure 26(*b*), we plot the dependence of 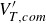 on the cluster’s bluntness index, again showing both primary and terminal values with supplementary means and frequency histograms included. As with the collective lateral velocity, the centre-of-mass offset (both postprimary and terminal) correlates weakly with the cluster’s bluntness index. This general relationship likely follows from a combination of increased ejection of bodies from blunter configurations and the inevitability of subsequent wake entrainment for these 13-sphere clusters. The terminal centre-of-mass velocity offset, however, shows some notable outliers, one of which occurs at 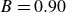 ( 
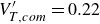
 ) and is the result of an exceptional seven-body subcluster. The survey-wide mean value of 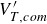 rises 54 % from 0.058 to 0.083 over the secondary separation phase, exceeding the increase experienced by the collective lateral velocity. This dynamic is consistent with the previous observation that the centre-of-mass offset is typically driven by wake entrainment events, a common feature of the secondary phase, whereby subclusters draw the centre of mass in their direction of travel. The associated frequency profiles are not readily categorized into recognizable distributions, which may indicate a lack of statistical convergence in the survey, geometrical constraints of the base 13-sphere configuration, and/or the inherent properties of the centre-of-mass motion itself. Nevertheless, the reduction in the mean centre-of-mass offset from the four-sphere survey (0.083 from 0.13) points to the bulk motion of more populous clusters aligning more closely with the primary axis of their prefragmented trajectories.

### 5.4. Central sphere dynamics

**Figure 27.**
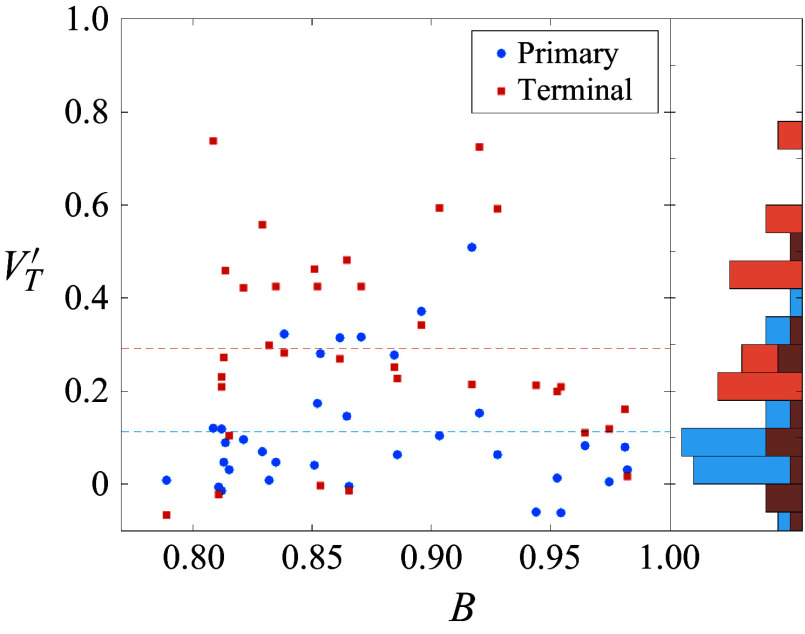
Primary and terminal separation velocities of internal spheres in the 13-sphere survey with mean values in dashed lines and frequency distributions shown to the right-hand side.

One aspect of the 13-sphere cluster dynamics that we have not yet considered is the presence of the central body initially located at the cluster’s centre of mass. An inspection of the cluster separation sequences, however, reveals that this central body plays an important role in the dispersion of fragments, particularly in the secondary phase. In figure 27, we thus present the lateral velocity of the central body following both the primary and secondary separation regimes versus the bluntness index, 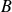 . We see that, with few exceptions, the central sphere accrues minimal lateral momentum over the course of the primary phase, which makes sense considering its position and the approximate isotropic nature of primary separation. A peak in the frequency distribution occurs near 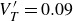 , with outlying examples typically occurring at intermediate values of 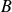 . The mean lateral velocity nearly triples between separation stages, however, rising from a modest value of 0.11 to 0.29, an indication of this particular sphere’s augmented aerodynamic significance in subcluster interactions. The terminal lateral velocities of the central body show no evident dependence on the cluster’s bluntness up to 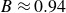 , but we note a rapid drop-off for higher bluntness indices, similar to the postprimary value. Given the unique nature of this body in the present cluster geometry, we must be cautious about drawing general conclusions for internal bodies in more populous clusters; nevertheless, it seems reasonable to extrapolate that initially shielded bodies may also play a pronounced role on secondary dynamics in such cases.

## 6. Conclusions

In this work, we have employed a coupled CFD–FEA framework to explore the separation of regular clusters of equally sized spheres in an inviscid, perfect-gas, Mach-20 flow, with the intention of deepening our physical understanding of the fragmentation of meteoritic bodies within the atmosphere. The spheres were released impulsively from three different configurations – touching pairs, tetrahedral four-sphere arrangements and face-centred-cubic 13-sphere clusters – at various initial orientations to the free stream and subsequently allowed to fly freely in response to the aerodynamic forces experienced. Surface contact and elastic collisions were accounted for through the finite-element component of the numerical framework.

The two-sphere survey revealed that, in addition to more familiar direct-repulsion and shock-surfing behaviour, a stable lifting configuration could develop for initially touching spheres over a limited range of skewed alignment angles. Lift coefficients of up to 0.22 for the pair could result, potentially leading to significantly enhanced lateral separation velocities. Indeed, such lifting pairs were found to arise in the larger-population clusters and could substantially modify the overall cluster dynamics, particularly at later times during the secondary separation phase.

For the four-sphere clusters, by binning the individual trajectories according to the initial polar angle of the sphere, it was found that this parameter was strongly correlated with the trajectory type and final lateral velocity. The binned mean lateral velocity increased roughly linearly with the polar angle measured from the axis of travel of the cluster, until reaching a maximum near 70 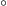 –80 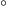 where shock surfing (and the extended lateral repulsion that results) was found to be most likely. The azimuthal angle, meanwhile, was found to change very little for most spheres, justifying the assumption of a purely lateral separation that is often invoked in modelling fragmentation events. For the bulk cluster dynamics, indices describing the initial cluster bluntness and asymmetry were introduced. Both the collective fragment separation velocity and the centre-of-mass offset velocity generally rose as the bluntness index was increased from small values; however, peak collective velocities were not attained for the bluntest configurations (for which the asymmetry index is necessarily low) but rather for moderately to highly blunt clusters that also exhibited significant asymmetry. Somewhat surprisingly, maximum centre-of-mass lateral velocities were not observed for the most asymmetric configurations, with clusters that had both moderate asymmetry and bluntness instead showing the largest values. These results may help to inform predictions of both the likely fall radius and bulk trajectory offsets of meteoroids that disrupt into a small number of fragments.

For the 13-sphere clusters, as in the four-sphere case, the initial cluster bluntness has a substantial effect on the individual sphere dynamics and associated flow field, especially during the primary (early) separation phase. Both the ultimate collective separation velocity (which increased with this larger population) and centre-of-mass offset (which decreased) showed a reduced sensitivity to initial orientation than for the four-sphere clusters, though increased variability was observed for the less blunt configurations. This points to a reduced influence of initial orientation for more populous clusters, at least for the regular parent geometries considered here. The individual fragment trajectories were once again found to be reasonably well-predicted by the initial sphere polar angle within the cluster, and the behaviour of the final lateral velocity as this parameter was varied showed a striking similarity to the four-sphere case. This consistency makes it plausible that a reduced-order statistical model of separation could predict the terminal trajectory of constituent fragments reasonably accurately without specifically treating the geometrical intricacies of the initial cluster, considerably simplifying the modelling of such fragmentation events.

The present study has deepened our understanding of the separation physics of limited-population clusters in high-speed flow, at least for the highly idealized geometries examined here. With the foundation provided by this work, a logical next step would be to relax some of the more stringent assumptions made here in a systematic way. For example, modifying the fragment geometries to include unequally sized and/or non-spherical bodies would bring the simulated configuration closer to a realistic fragmented meteoroid, though at the expense of a vastly increased parameter space. Considering irregular parent geometries would also be a natural extension of this work and quite feasible within the present computational framework.

## Supporting information

Whalen et al. supplementary material 1 Whalen et al. supplementary material

Whalen et al. supplementary material 2 Whalen et al. supplementary material

Whalen et al. supplementary material 3 Whalen et al. supplementary material

Whalen et al. supplementary material 4 Whalen et al. supplementary material

Whalen et al. supplementary material 5 Whalen et al. supplementary material

Whalen et al. supplementary material 6 Whalen et al. supplementary material

Whalen et al. supplementary material 7 Whalen et al. supplementary material

Whalen et al. supplementary material 8 Whalen et al. supplementary material
